# Advances in Molecular Mechanisms of Kidney Disease: Integrating Renal Tumorigenesis of Hereditary Cancer Syndrome

**DOI:** 10.3390/ijms25169060

**Published:** 2024-08-21

**Authors:** Rossella Cicchetti, Martina Basconi, Giulio Litterio, Marco Mascitti, Flavia Tamborino, Angelo Orsini, Alessio Digiacomo, Matteo Ferro, Luigi Schips, Michele Marchioni

**Affiliations:** 1 Department of Medical Oral and Biotechnological Science, Università degli Studi “G. d’Annunzio” of Chieti, 66100 Chieti, Italy; rossella.cicchetti3@gmail.com (R.C.); basconimartina1@gmail.com (M.B.); giulio.litterio@gmail.com (G.L.); mar.mascitti@gmail.com (M.M.); flavia.tamborino96@gmail.com (F.T.); orsini.ang@gmail.com (A.O.); digiacomoale90@gmail.com (A.D.); mic.marchioni@gmail.com (M.M.); 2 Division of Urology, European Institute of Oncology, Istituto di Ricovero e Cura a Carattere Scientifico (IRCCS), 20141 Milan, Italy; matteo.ferro@ieo.it

**Keywords:** kidney cancer, hereditary cancer syndromes, von Hippel–Lindau disease, Birt–Hogg–Dubè syndrome, succinate dehydrogenase-B mutation, tuberous sclerosis complex, hereditary papillary renal cell carcinoma, fumarate hydratase deficiency, BAP1 tumor predisposition syndrome

## Abstract

Renal cell carcinoma (RCC) comprises various histologically distinct subtypes, each characterized by specific genetic alterations, necessitating individualized management and treatment strategies for each subtype. An exhaustive search of the PubMed database was conducted without any filters or restrictions. Inclusion criteria encompassed original English articles focusing on molecular mechanisms of kidney cancer. On the other hand, all non-original articles and articles published in any language other than English were excluded. Hereditary kidney cancer represents 5–8% of all kidney cancer cases and is associated with syndromes such as von Hippel–Lindau syndrome, Birt–Hogg–Dubè syndrome, succinate dehydrogenase-deficient renal cell cancer syndrome, tuberous sclerosis complex, hereditary papillary renal cell carcinoma, fumarate hydratase deficiency syndrome, BAP1 tumor predisposition syndrome, and other uncommon hereditary cancer syndromes. These conditions are characterized by distinct genetic mutations and related extra-renal symptoms. The majority of renal cell carcinoma predispositions stem from loss-of-function mutations in tumor suppressor genes. These mutations promote malignant advancement through the somatic inactivation of the remaining allele. This review aims to elucidate the main molecular mechanisms underlying the pathophysiology of major syndromes associated with renal cell carcinoma. By providing a comprehensive overview, it aims to facilitate early diagnosis and to highlight the principal therapeutic options available.

## 1. Introduction

Renal cell carcinoma (RCC) originates from the renal parenchyma (i.e., renal epithelium) [1]. Globally, renal cell carcinoma (RCC) stands as the sixth most commonly diagnosed cancer in men and the eighth in women, constituting 5% and 3% of all cancer diagnoses, respectively. In the year 2020, approximately 431.288 new cases of kidney cancer (KC) were reported globally. Despite most of these identified masses being small tumors, a considerable number of patients were diagnosed with locally advanced disease. Shockingly, as many as 17% of individuals are found to have distant metastases upon initial diagnosis [2]. Sporadic RCC is a condition predominantly found in the elderly population, with an average diagnosis age of 64 years in the United States. When RCC is diagnosed at a younger age (≤46 years), the possibility of an underlying hereditary kidney cancer should be considered, which accounts for 3–5% of all RCC cases [3].

Hereditary kidney cancer represents 5–8% of all kidney cancer cases. The majority of hereditary cancer syndromes (HCSs) are inherited in an autosomal dominant manner. In the most prevalent scenario, individuals carry a cancer-associated pathogenic variant (PV), either inherited from a parent or arising spontaneously (de novo), a concept known as ‘the two-hit mechanism’, proposed by Alfred G. Knudson. Based on the “two-hit” model, a dominantly inherited predisposition to cancer involves a germline mutation, with tumorigenesis necessitating an additional somatic mutation. In the case of non-hereditary cancer of the same type, both necessary mutations are somatic [4]. This PV is present in every cell of the body but typically does not result in observable effects due to the presence of a functional second allele of the same gene. Cancer initiation in hereditary kidney-related syndromes (HCSs) often occurs when the remaining functional allele of the gene is somatically inactivated. This inactivation can happen through various mechanisms, including deletion (leading to loss of heterozygosity; LOH), point mutation, or epigenetic silencing [5,6,7]. Most HCSs are not exclusively defined by renal malignancies but also affect other organs. Among the most well-known renal HCSs are fumarate hydratase tumor predisposition syndrome (FHTPS, previously known as hereditary leiomyomatosis and renal cell carcinoma (HLRCC)), von Hippel–Lindau (VHL) disease, and hereditary papillary renal cell carcinoma syndrome (HPRCC). Additionally, kidney involvement is frequently observed in conditions such as Birt–Hogg–Dubé syndrome, tuberous sclerosis, and BAP1 tumor predisposition syndrome, among others. Some data also suggest that Cowden syndrome, Lynch syndrome, CHEK2 germline mutations, and others may elevate the risk of renal malignancies [6].

In this review, we explain the main molecular mechanisms now known in order to understand the pathophysiology of the principal syndromes regarding renal cell cancer to provide a complete view and how to make an early diagnosis, as well as the principal therapies available.

## 2. Hereditary Cancer Syndromes: Main Molecular Mechanisms

### 2.1. Von Hippel–Lindau Disease

Since the von Hippel–Lindau (VHL) disease tumor suppressor gene VHL was identified in 1993 as the genetic basis for a rare disorder, the von Hippel–Lindau (VHL) syndrome (OMIM 193300) has been an autosomal dominant hereditary condition characterized by a predisposition to tumors [8]. VHL first manifests in the second decade of life. Individuals carrying a disease-causing germline variant in the VHL gene face the risk of developing both benign and malignant tumors across multiple organs [9]. The incidence of VHL ranges from 1 in 36,000 to 1 in 45,000 live births. VHL has a point prevalence of 1 in 38,000 to 1 in 91,000 persons [10,11].

Treacher Collins (1894) and von Hippel described familial retinal hemangioblastomas in pathological specimens from three enucleations of two siblings (Melmon and Rosen, 1964). Lindau recognized the association between retinal angiomas and cerebellar hemangioblastoma. Although Lindau also noted the presence of renal tumors and cysts, they were initially considered benign. Melmon and Rosen conducted a literature review on what would later be termed VHL disease and proposed clinical diagnostic criteria. The term “von Hippel–Lindau disease” was first coined in 1936 and has been widely used since the 1970s [12,13].

#### 2.1.1. Molecular Mechanism

Linkage studies indicate that the VHL gene is a tumor suppressor gene on chromosome 3p25–26. The gene consists of three exons and encodes the VHL protein, a glycan-anchored membrane protein responsible for signal transduction [8].

Initially, a germline VHL mutation leads to a faulty allele present in all cells throughout the body (first hit). However, for tumor formation to occur, a second somatic event is necessary (commonly referred to as the second hit or loss of heterozygosity) [14]. The majority of VHL patients inherit a germline mutation from an affected parent, along with a wild-type allele from the unaffected parent [10]. Mutation analysis enables the presymptomatic identification of affected family members; those who do not inherit the gene do not require monitoring. The coding sequence of the VHL gene consists of three exons, and there are two mRNA isoforms, depending on the presence or absence of exon 2. Tumors develop following the loss or inactivation of the wild-type allele in a cell.

Missense mutations (27–38%) and nonsense mutations (13–27%) represent the predominant types of germline mutations. The majority of the remaining germline mutations consist of large deletions (9–20%), microdeletions (10%), and rearrangements (25%), while insertions or splice-site mutations are infrequent [15]. Somatic mutation events that result in homozygous VHL allele inactivation are key drivers of tumorigenesis. These “second-hit” events commonly involve allelic loss (49%) or hypermethylation (35%) of the wild-type allele [16]. Somatic inactivation of the wild-type allele may also arise from point mutations, particularly in cases of large 3p deletions as the initial event [17]. Approximately 20% of patients have large germline mutations detectable by Southern blot analysis, 27% have missense mutations, and 27% have nonsense or frameshift mutations. In about 20% of VHL families, no deletion or mutation can be detected. Families may be classified by the presence (type 2; 7% to 20% of families) or absence (type 1) of pheochromocytomas. Most type 2 families have missense mutations, whereas most type 1 families are affected by deletions or premature termination mutations [18].

Recent research has revealed that the product of the von Hippel–Lindau (VHL) tumor suppressor gene, which is deactivated in VHL disease and sporadic clear cell renal carcinomas, targets the transcription elongation complex known as elongin.

**Figure 1:**
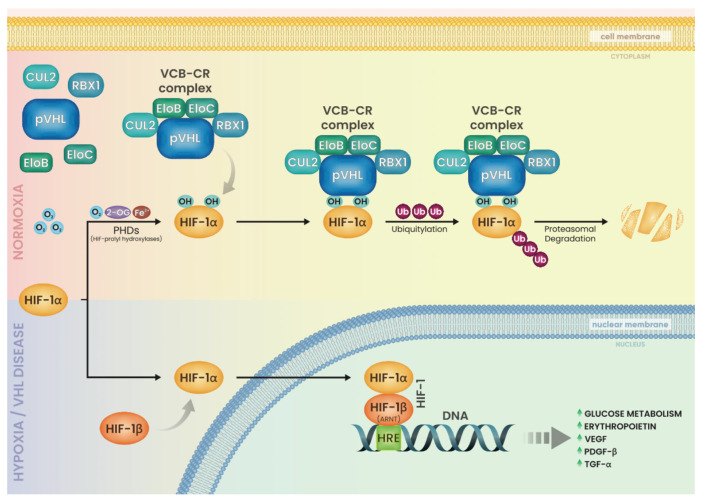
Molecular mechanism of von Hippen–Lindau disease [1,2,3].

The VHL gene is responsible for encoding the VHL protein (pVHL), which consists of 213 amino acid residues and weighs between 24 and 30 kDa. Additionally, a smaller isoform weighing 19 kDa is present in various tissues. This particular form of pVHL is generated through alternative translation initiation from codon 54 [12]. Both forms are capable of suppressing tumor growth [19]. Von Hippel–Lindau (VHL) disease arises due to inactivating mutations located between codon 54 and the carboxy-terminal region, impacting the functionality of both protein isoforms [20]. The primary localization of pVHL is within the cytosol, yet it exhibits dynamic movement, allowing it to shuttle into and out of the nucleus [21]. The most well-understood role of pVHL is its function in targeting hypoxia-inducible factor (HIF) for degradation via the proteasome [22]. HIF is a heterodimeric transcription factor consisting of an unstable alpha (α) subunit and a stable beta (β) subunit. Three human HIF-α genes have been identified thus far [23]. HIF-3α appears to be involved in a feedback mechanism that regulates the activity of HIF-1α; pVHL binds to elongin C, which in turn binds to elongin B and CUL2 to form the VCB-CUL2 complex. Under normal oxygen levels, the VCB-CUL2 complex associates with HIF-α, inducing polyubiquitination that marks it for degradation by the proteasome [24] (Figure 1).

**Figure 2:**
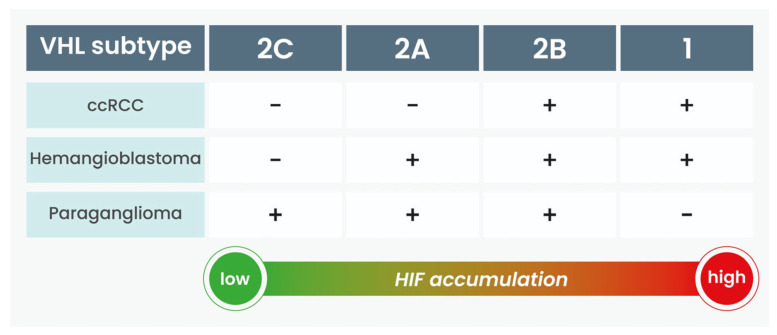
Mutant VHL alleles associated with type 1, type 2A, type 2B, and type 2C VHL disease exhibit varying degrees of HIF deregulation [4].

HIF-α undergoes hydroxylation within the oxygen-dependent degradation domain (ODD) in normoxic conditions. This hydroxylation facilitates binding with pVHL and the VCB-CUL2 complex, triggering ubiquitination and subsequent proteasomal degradation. Conversely, in hypoxic environments HIF-α interacts with the cyclic AMP response element-binding protein and the transcriptional coactivator p300 to activate the transcription of HIF-regulated genes and as a consequence increase glucose metabolism, VEGF, PDGF-β, erythropoietin, and TGF-α. In instances where pVHL is inactive, tumorigenesis may ensue through either HIF-mediated pathways or through direct consequences of pVHL malfunction [13,23,25]. Initial studies focused on familial cases, revealing a clustering of manifestations among VHL carriers. The classification of VHL into types 1, 2A, 2B, and 2C originated from the observation of clinical phenotypic patterns. Later studies proved that mutant VHL alleles associated with type 1, type 2A, type 2B, or type 2C VHL disease exhibit varying degrees of HIF deregulation. Relative HIF levels are highest in type 1 and lowest in type 2C, following this order: type 1 > type 2B > type 2A > type 2C. In relation to the expression of HIF, we observe a distinct clinical manifestation. Type 1 disease is probably induced by mutations resulting in severe disruption of protein activity, such as deletions, nonsense mutations, and other microdeletions/insertions. Type 2 disease nearly always (78–96%) is associated with missense mutations [26] (Figure 2).

Typically, VHL is distinguished by the development of numerous benign and malignant tumors, alongside cysts affecting multiple organs. Those affected commonly experience the emergence of retinal and central nervous system hemangioblastomas, clear cell renal cell carcinomas (RCC), pheochromocytomas, pancreatic neuroendocrine tumors, and endolymphatic sac tumors (ELSTs). VHL disease is a complex multisystem disorder that requires collaboration among various medical specialties. Coordinating care for VHL families can be challenging but is crucial to reduce preventable morbidity and mortality [13].

#### 2.1.2. Focus on Kidney Cancer

In patients diagnosed with VHL syndrome, clear cell renal cell carcinoma (ccRCC) consistently emerges as the predominant histological subtype of kidney cancer. Reported incidence rates of ccRCC range from 24% to 45% across various retrospective series [27]. The onset of ccRCC in individuals with VHL syndrome typically occurs around 20 years earlier than the average age at which sporadic RCC is diagnosed. ccRCC in VHL syndrome is characterized by early onset, the presence of multiple tumors in both kidneys, and a tendency for recurrence (Table 1). On average, ccRCC is diagnosed in VHL syndrome patients at the age of 39. Recent research suggests that the annual growth rate of renal tumors in those with VHL deficiency is 0.37 cm/year, slightly lower than that observed in sporadic renal tumors (0.43 cm/year) [28].

Furthermore, CT or MRI scans enable urologists and oncologists to determine the total number of tumors and cysts, as well as the size, location, and clinical stage of the tumors. Since these factors influence decisions regarding surveillance or active treatment, utilizing contrast-enhanced CT or MRI is often recommended for standardizing follow-up care (Table 2).

Because nearly 80% of VHL cases are familial, with the genetic mutation inherited in an autosomal dominant manner, genetic testing is crucial for assessing the risk to other family members [29].

Given that RCC is a leading cause of morbidity and mortality, its management is a crucial prognostic factor for individuals with VHL syndrome. The approach involves active surveillance until the largest tumor reaches 3 cm in size, at which point nephron-sparing surgery is recommended to reduce the risk of metastasis and preserve kidney function. During surgery, all tumors and cysts in the affected kidney should be removed, regardless of their size. For small cysts that cannot be excised, unroofing and fulguration of the cyst base are advised, as the cyst walls in VHL patients, lined with clear cells, have the potential to become malignant within 3–7 years. If the tumor’s size or location is not suitable for NSS, a radical nephrectomy should be performed. Even when all tumors and cysts are removed in a single-stage NSS, recurrent tumors may still develop in the same kidney after surgery in patients with VHL syndrome [30]. Therefore, the objective of NSS in VHL syndrome is not to eliminate the disease but to manage it effectively [31]. In recent developments, belzutifan, a pioneering inhibitor targeting HIF-2α, exhibited notable anti-tumor efficacy in individuals with VHL syndrome-related tumors, including renal cell carcinoma (RCC) [32]. Belzutifan represents an innovative small molecule designed to address HIF-2α, which experiences heightened expression in VHL disease due to the absence of its VHL protein-dependent ubiquitination and degradation. By inhibiting HIF-2α, its transcriptional function is hindered, resulting in the suppression of genes associated with angiogenesis, cell proliferation, and immune evasion [33] (Table 2).

### 2.2. Birt–Hogg–Dubé Syndrome

Birt–Hogg–Dubé syndrome (BHD) is a hereditary condition marked by cutaneous lesions (acrochordons, fibrofolliculomas, and trichodiscomas), pulmonary cysts, pneumothorax, and renal cysts and tumors.

In 1977, Birt, Hogg, and Dubé investigated a Canadian family with thyroid cancers, identifying fibrofollicular skin tumors inherited in an autosomal dominant pattern. German dermatologists Hornstein and Knickenberg reported a case involving a middle-aged woman and her brother with multiple perifollicular fibromas and intestinal polyps, suggesting inheritance from their father, who had renal and lung cysts and skin tumors. They termed it “a cutaneo-intestinal syndrome sui generis”. Both reports discussed a potential unique hereditary disorder and referenced earlier studies. Although Birt et al. noted histopathological differences, subsequent analysis showed that skin tumors in BHD patients represent a spectrum of the same tumor. Happle proposed renaming the syndrome “Hornstein–BHD”. Renaming raises acknowledgment issues, with Knickenberg deserving recognition. In 2001, two research groups identified the chromosomal location of the gene responsible for BHD, later designated as FLCN (folliculin).

Birt–Hogg–Dubé syndrome is an inherited condition characterized by a mutation in the FLCN (folliculin) gene, located on chromosome 17p11. The estimated prevalence of the syndrome is around 1 in every 200,000 individuals, with approximately 600 families carrying FLCN mutations identified thus far. The typical age for diagnosing renal cell carcinoma (RCC) in BHD patients falls within the range of 46 to 56 years, with an average age of 50.7 years [34].

#### 2.2.1. Molecular Mechanism

Much of the current knowledge concerning BHD-associated renal cell carcinoma (RCC) has been gleaned from studies employing BHD animal models and a cell line derived from BHD kidney tumors. These investigations have linked FLCN to numerous cellular processes, including mitochondrial biogenesis, stress response, autophagy, membrane trafficking, stem cell pluripotency, and ciliogenesis. The FLCN protein interacts with various regulatory factors, such as the mTOR, AMPK, HIF1, TGF-β, and Wnt pathways.

In Baba M. et al.’s research, the loss of gene function is associated with the dysregulation of mTOR, a key regulator of cell growth and size by stimulating protein synthesis. BHD syndrome, categorized as a hamartoma disorder, shares phenotypic resemblances with tuberous sclerosis complex (TSC), prompting speculation that BHD may intersect with the pathways signaling through mTOR. To elucidate FLCN’s function, they conducted a search for interacting proteins through co-immunoprecipitation. They identified a 130 kDa FLCN-interacting protein, FNIP1, and demonstrated its interaction with AMPK, a crucial protein in nutrient/energy sensing. Their investigations revealed that FLCN undergoes phosphorylation, facilitated by FNIP1, and is suppressed under conditions inhibiting mTOR. Moreover, they observed that treatment with an AMPK inhibitor reduced FNIP1 phosphorylation, diminished FNIP1 expression levels, and led to FLCN dephosphorylation. Based on these observations, they proposed a hypothesis suggesting that FNIP1 and FLCN could function as downstream effectors of mTOR and AMPK, modulating energy/nutrient-sensing signaling pathways through an as-yet-undisclosed molecular mechanism [35].

According to research conducted by Possik E. et al., it was demonstrated that FLCN serves as an evolutionarily conserved negative modulator of AMPK. Through experiments using both Caenorhabditis elegans and mammalian cells, they demonstrated that the absence of FLCN triggers persistent activation of AMPK. This activation leads to the initiation of autophagy, inhibition of apoptosis, enhancement of cellular bioenergetics, and the acquisition of resistance to various energy-depleting stresses, including oxidative stress, heat, and anoxia. Furthermore, the investigation indicates that the activation of AMPK due to FLCN loss occurs independently of the cellular energy status, suggesting that FLCN governs the energy-sensing capability of AMPK. Collectively, the analysis of the data suggests that FLCN functions as an evolutionarily conserved regulator of AMPK signaling, potentially acting as a tumor suppressor by exerting negative control over AMPK activity [36]. Two studies have demonstrated that the absence of folliculin results in mTOR inhibition and that it plays a role in nutrient sensing by functioning as a GTPase-activating enzyme for the RAG GTPases. It is plausible that FLCN operates within two distinct complexes. Under starvation conditions, it associates with RAGs, resulting in mTOR inhibition, whereas under normal conditions, FLCN would interact with AMPK, suppressing its activity. However, it remains unclear how the observed mTOR inhibition due to FLCN loss could lead to tumorigenesis, given that mTOR has been shown to be hyperactivated in tumors of BHD patients and FLCN-deficient mice [37,38]. Previous research in yeast, C. elegans, and mammalian cells has shown that LGG-1-II (or LC3-II) undergoes degradation inside autolysosomes, with the GFP fragment being resistant to degradation and accumulating when autophagy is activated [39,40]. Possik et al.’s research conducted western blot analysis on protein extracts from wild-type and flcn-1 mutants to assess GFP-LGG-1 levels and cleavage. The results indicated that both cleaved LGG-1-II and released GFP were elevated in flcn-1(ok975) mutants, suggesting increased autophagic activity. Recent findings have shown that AMPK directly induces autophagy in mammals by phosphorylating autophagy-related proteins such as ULK-1, VPS-34, and BEC-1 [41]. Additionally, the absence of aak-2 reduces autophagy in daf-2 mutant animals, while overexpression of aak-2 promotes autophagy [42]. In C. elegans, inhibiting autophagy genes has been shown to decrease survival under conditions such as anoxia, starvation, and pathogen exposure. These findings collectively indicate that flcn-1 loss triggers autophagy, which is crucial for the stress resistance mediated by flcn-1 [43].

The mechanisms that sustain the self-renewal of mouse embryonic stem cells (ESCs) are well understood, whereas the process governing the transition from pluripotency to differentiation remains unclear. In an investigation led by Betschinger J. et al., through a comprehensive small interfering RNA (siRNA) screen, they discovered that the knockdown of the tumor suppressors Folliculin (Flcn) and Tsc2 prevents ESCs from committing to differentiation. Tsc2 acts upstream of the mammalian target of rapamycin (mTOR), while Flcn functions downstream and independently. Flcn, together with its interacting partners Fnip1 and Fnip2, promotes differentiation by controlling the nuclear localization and activity of the bHLH transcription factor Tfe3. Conversely, increased nuclear Tfe3 expression enables ESCs to resist differentiation stimuli. Genome-wide analyses revealed that Tfe3 directly influences the pluripotency circuitry by regulating the transcription of Esrrb, a core pluripotency factor. These findings highlight an intrinsic mechanism for destabilizing ground-state pluripotency to facilitate lineage commitment. Additionally, the relocation of Tfe3 at specific developmental stages suggests that Flcn-Fnip1/2 contributes to the progression of pluripotent epiblast development in vivo [44].

In a separate study by Mathieu et al. in human ESCs, it was observed that the absence of FLCN leads to the exclusive nuclear localization of TFE3, resulting in the activation of its target genes. While TFE3 has been found to regulate the transcription of the core pluripotency factor Esrrb in mouse ESCs, the expression of Esbrrb is low in human ESC lines, indicating differences in TFE3 targets between species. Knocking out Esbrrb in naïve FLCN-deficient human ESCs did not significantly modify the expression of naïve markers during the transition from naïve to primed states. Several TFE3 target genes, including WNT ligands and pathway targets, were upregulated in FLCN-deficient human ESCs, suggesting activation of the WNT pathway. Since WNT expression has been shown to inhibit the transition from naïve to primed ESCs, the study proposed that TFE3-mediated WNT pathway activation in FLCN-deficient human ESCs prevents these cells from exiting the naïve state. This hypothesis was confirmed by demonstrating that a WNT inhibitor could rescue the phenotype of FLCN-deficient cells [45].

In accordance with the study conducted by Di Malta et al., the mechanistic Target of Rapamycin Complex 1 (mTORC1) is recruited to the lysosome through Rag GTPases and governs anabolic pathways in response to nutrient stimuli. Analysis of data indicated that MiT/TFE transcription factors, key regulators of lysosomal and melanosomal biogenesis as well as autophagy, manage mTORC1 lysosomal recruitment and function by directly modulating RagD expression. This mechanism in mice facilitated adaptation to food availability following starvation and physical exertion, and significantly contributed to cancer progression. Elevated expression of MiT/TFE oncogenes in cellular and tissue contexts, including renal cell carcinoma, pancreatic ductal adenocarcinoma, and melanoma, instigated RagD-mediated mTORC1 activation, leading to increased cell proliferation and tumor growth. Thus, this transcriptional regulatory process enables cellular adjustment to nutrient availability and sustains the metabolically demanding nature of cancer cells [46].

In research conducted by Dunlop E.A. et al., autophagy plays a crucial role in maintaining cellular equilibrium by eliminating damaged organelles and large molecules. Despite the known involvement of the autophagy kinase ULK1 in this process, the precise mechanisms underlying its action are still being elucidated, and only a few ULK1 substrates have been identified thus far. Their study reveals that the absence of FLCN results in a moderate impairment of the basal autophagic flux, whereas reintroduction of FLCN restores autophagy. Additionally, they identify three previously unknown phosphorylation sites within FLCN (Ser406, Ser537, and Ser542) induced by ULK1 overexpression, indicating regulation of the FLCN complex by ULK1. Furthermore, their investigation demonstrates that FLCN interacts with GABA(A) receptor-associated protein (GABARAP), a key component of the autophagy machinery. The association between FLCN and GABARAP is influenced by the presence of folliculin-interacting protein (FNIP)-1 or FNIP2 and further modulated by ULK1. Examination of chromophobe and clear cell tumors from a patient with BHD revealed elevated levels of GABARAP, sequestome 1 (SQSTM1), and microtubule-associated protein 1 light chain 3 (MAP1LC3B), suggesting impaired autophagy in BHD-associated renal tumors [47].

In a study by J.A. Klomp et al., the predominant molecular characteristic observed in kidney tumors arising from Birt–Hogg–Dubé syndrome (BHDS) was the heightened expression of genes related to mitochondria and oxidative phosphorylation (OXPHOS). This expression pattern in mitochondria was linked to the dysregulation of the PGC-1a-TFAM signaling pathway. Furthermore, the absence of FLCN expression in different tumor types was correlated with elevated expression of nuclear mitochondrial genes. The dysregulation of the PGC-1a-TFAM signaling pathway is particularly notable in kidney tumors bearing FLCN mutations and in tumors originating from other organs with comparatively diminished FLCN expression levels. These findings align with the recently identified interaction between FLCN and AMPK, endorsing a framework wherein FLCN serves as a modulator of mitochondrial functionality [48].

In a study by N.H. Luijten et al., it is indicated that FLCN is localized in both motile and non-motile cilia, centrosomes, and the mitotic spindle. Alterations in FLCN levels can impact the initiation of ciliogenesis without completely inhibiting it. In three-dimensional culture, abnormal FLCN expression disrupts the polarized growth of kidney cells and interferes with canonical Wnt signaling. Their findings suggest that mutations in FLCN associated with BHD may retain partial functionality, indicating that certain BHD symptoms could result from abnormal FLCN levels rather than complete loss of function. Consequently, mutant forms of FLCN are detected in renal carcinoma associated with BHD, suggesting that BHD represents a novel ciliopathy characterized, in part, by aberrant ciliogenesis and canonical Wnt signaling [49].

However, the precise mechanism underlying how FLCN loss triggers tumorigenesis remains largely unknown. Conflicting findings, such as contradictory effects of FLCN on mTOR signaling, and the diverse range of processes associated with FLCN loss, pose challenges to fully understanding the pathways through which FLCN suppresses renal tumorigenesis.

**Figure 3:**
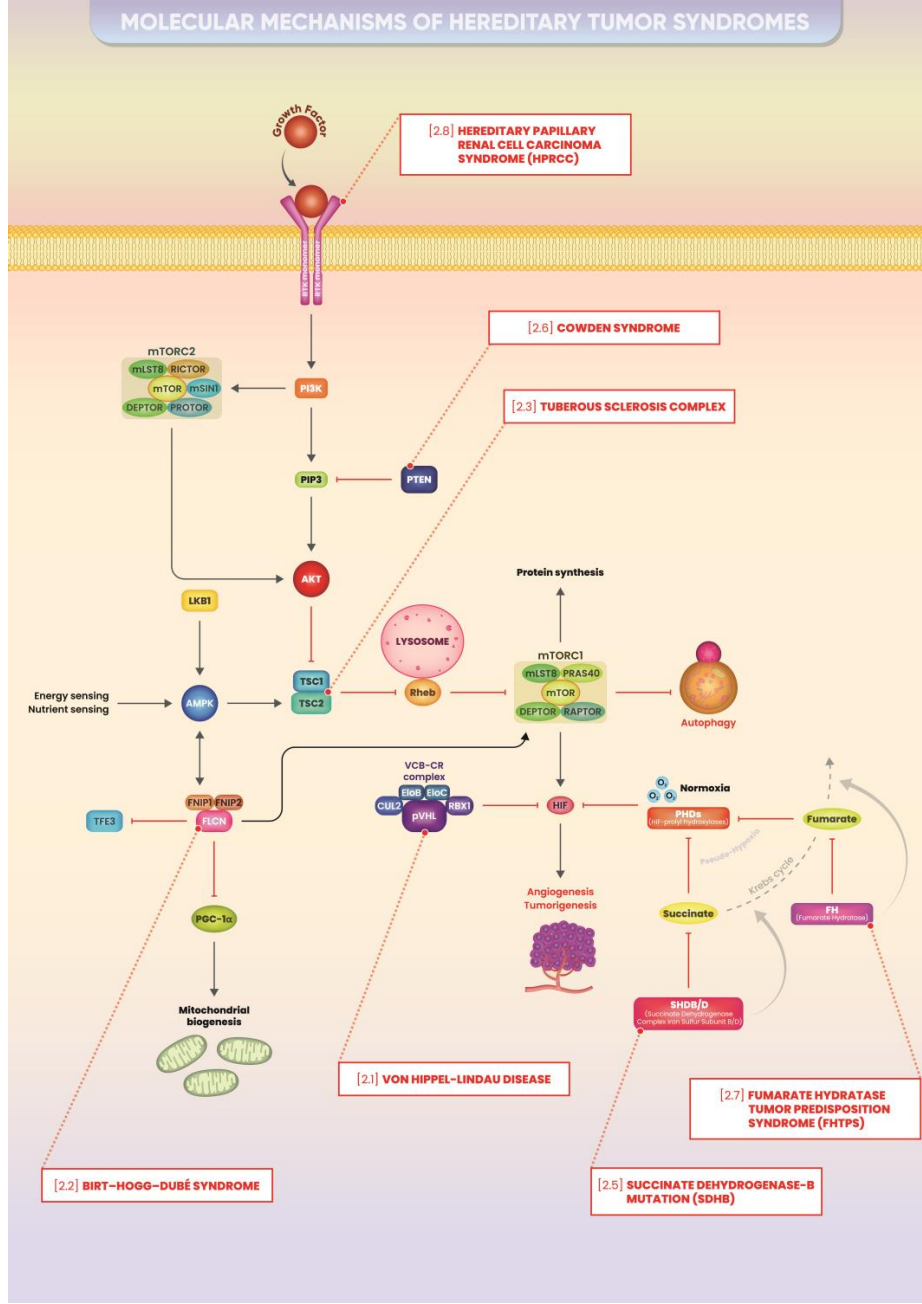
Summary of principal molecular mechanisms of tumorigenesis of hereditary cancer syndrome.

A recent publication by Glykofridis et al. has outlined the molecular and cellular outcomes associated with the deletion of FLCN or its binding partners FNIP1/FNIP2 in a human renal proximal tubular epithelial cell model (RPTEC/TERT1), which mirrors the cell types implicated in RCC [50]. Through RNA sequencing (RNAseq) and proteomic analyses, followed by pathway assessments and exploration of regulatory promoter motifs in genes with altered expression, it was uncovered that the absence of FLCN leads to two distinct transcriptional patterns. The first pattern is characterized by genes regulated by E-box elements and validates TFE3 as a key target of the FLCN-FNIP1/2 axis [36,51,52]. Additionally, it has been found that the loss of FLCN-FNIP1/2 triggers the activation of a group of genes controlled by interferon-stimulated response elements (ISREs). This activation of ISRE-associated genes is mediated by the increased expression of STAT1 and STAT2, providing insight into the paradoxical decrease in cellular proliferation following FLCN tumor suppressor loss. The study proposed that TFE3 and STAT1/2 serve as the primary, independent transcriptional regulators of FLCN-FNIP1/2 deficiency in human renal epithelial cells. This theory is consistent with the finding that renal tumors propelled by TFE3 activation exhibit more aggressive behavior compared to the slowly developing BHD tumors. It is speculated that BHD tumors can be classified into two separate categories, each showing either high or low levels of IFN signatures and/or STAT2 expression, depending on their growth patterns [53]. Regarding potential target genes regulated by TFE3 that promote the transformation of renal epithelial cells, it is evident that in the absence of FLCN, notably GPNMB, RRAGD, ASAH1, and FNIP2, the latter through an apparent feedback mechanism, are significantly upregulated. Similarly, in renal cells deficient in FNIP1 and FNIP2, GPNMB and RRAGD are also markedly induced. This highlights the potential significance of GPNMB and RRAGD as favorable biomarkers for FLCN inactivation, as seen in BHD tumors [54]. GPNMB is a membrane-bound protein frequently elevated across various tumor types, including lung and renal cancers. Notably, therapeutic targeting of GPNMB is feasible using antibody–drug conjugates, such as glembatumumab vedotin, which are undergoing clinical trials for cancer treatment [55]. This presents an opportunity to explore the efficacy of glembatumumab in treating BHD tumors. Additionally, RRAGD emerges as a potential oncogenic target gene regulated by TFE3, previously linked to FLCN loss [46] (Figure 3).

#### 2.2.2. Focus on Kidney Cancer

Birt–Hogg–Dubé syndrome (BHD) is a hereditary condition characterized by kidney cancer, primarily caused by mono-allelic germline loss-of-function mutations in the Folliculin (FLCN) gene, which is essential and conserved. Individuals with BHD have a lifetime risk of developing renal cell carcinoma (RCC) approximately ten times higher than the general population [56,57]. They are prone to bilateral and multifocal renal tumors and require regular surveillance via renal imaging for early detection and effective treatment before metastasis occurs [58]. The development of kidney cancer in BHD patients typically involves loss of heterozygosity through gene silencing or inactivating somatic mutations of the wild-type FLCN allele [59].

Unlike in von Hippel–Lindau (VHL) disease, where renal tumors predominantly manifest as clear cell carcinomas, the renal tumors associated with BHD exhibit a variety of histological subtypes. Approximately 50–67% of tumors are hybrid oncocytic–chromophobe type, 23–34% are chromophobe tumors [60], 7–9% are clear cell, 3–5% are oncocytomas, and around 2% are papillary renal cancer [61]. Renal tumors in BHD are more indolent compared to those in VHL, with an observed growth rate averaging 0.11 cm/year [62]. In individuals with BHD, the characteristic renal lesions typically present themselves as predominantly solid masses, contrasting with the VHL syndrome where lesions often exhibit a combination of solid and cystic components. These BHD-associated lesions demonstrate homogeneous enhancement on imaging compared to the renal cortex, distinguishing them from papillary tumors. Occasionally, calcifications may also be observed within these lesions [63]. Oncocytomas, a subtype of renal tumors associated with BHD, are often identified by the presence of a central stellate scar [64] (Table 1).

Patients with a familial history of FLCN mutation or exhibiting clinical features indicative of BHD undergo genetic screening [65]. Birt–Hogg–Dubé (BHD) syndrome is a complex disease that affects multiple systems, necessitating multidisciplinary screening approaches involving urologists, pulmonologists, dermatologists, and geneticists. Patients with family history of FLCN mutation or with other clinical features suggestive of the syndrome (i.e., fibrofolliculomas or history of spontaneous pneumothorax with multiple pulmonary cysts) are recommended to undergo genetic screening for BHD, with genetic counseling for their family. After undergoing chest CT scans to assess pulmonary involvement, skin lesions are examined by a dermatologist. Urological guidelines recommend that for cancer risk management in Birt–Hogg–Dubé syndrome, a baseline abdominal MRI or CT should be performed at age 20. If no abnormalities are observed, follow-up imaging should consist of either MRI every 2 years or high-resolution ultrasound throughout the individual’s lifetime. While abdominal MRI is the preferred imaging technique, high-resolution ultrasound serves as an acceptable alternative if MRI is not accessible. Research was conducted by Johannesma PC. on 199 patients with Birt–Hogg–Dubé syndrome in two Dutch hospitals until June 2014. Using ultrasound (US), 9 out of 18 tumors were not detected in the cohort. All missed tumors were smaller than 3 cm, with the largest being 27 mm. The histology of the undetected tumors was similar to that of the detected ones, indicating that histology is not a significant factor in the likelihood of missing a tumor on US. Consistent with the current literature, no metastatic disease occurred in patients with renal cell carcinoma (RCC) smaller than 3 cm. These findings highlight the importance of early diagnosis of BHD and initiating screening from age 20 or upon diagnosis of BHD. All four patients with metastatic RCC in the cohort developed the disease before being aware of their BHD status, and two of them were only in their twenties. Since most RCCs were diagnosed during initial screening and BHD-related RCCs are known to have an indolent nature, it is likely that some tumors in the cohort could have been detected earlier if screening had been conducted [58]. Subsequent management depends on the size and growth rate of the lesion [63] (Table 2).

Treatment protocols for renal tumors in BHD syndrome closely resemble those for von Hippel–Lindau (VHL) syndrome. When a dominant lesion reaches 3 cm, surgical resection, preferably via nephron-sparing surgery (NSS) utilizing enucleation, should be performed to preserve maximum renal parenchyma [66]. Metastasis in BHD is rare; however, when present, it is typically associated with large clear cell or papillary subtype tumors. Ablation therapy, akin to VHL management, is generally considered for patients unfit for surgery with solitary tumors measuring less than 3 cm in diameter [67].

Preclinical studies have indicated that loss of folliculin results in the upregulation of the mTOR pathway. Although mTOR inhibitors, such as everolimus, have shown some efficacy in treating metastatic BHD, they have not been effective in addressing fibrofolliculomas [68] (Table 2).

### 2.3. Tuberous Sclerosis Complex

Tuberous sclerosis complex (TSC) is a genetic disorder with autosomal dominant inheritance that affects various systems, including the nervous system (subependymal giant cell astrocytomas, cerebral cortical tubers), heart (rhabdomyomas), lung (lymphangioleiomyomatosis or LAM), skin (cutaneous and subungual angiofibromas, shagreen patch) and kidneys, with some patients developing seizures or mental retardation. It manifests with hamartomas in multiple organs and has been known by different names such as Pringle–Bourneville phacomatosis.

Its description dates back to the 19th century (in 1862) when Virchow and Von Recklinghausen first identified hamartomas in the brain and heart of patients with seizures and mental retardation. Bourneville later correlated the cutaneous manifestations with other clinical symptoms, leading to the formalization of the syndrome in the early 20th century. Campbell and Vogt further characterized TSC by establishing the triad of mental retardation, epilepsy, and Pringle-type sebaceous adenoma [69].

Diagnostic criteria for tuberous sclerosis were defined in 1998 and were later reviewed and updated during the second International Tuberous Sclerosis Complex Consensus Conference in 2012, which aimed to provide recommendations for diagnosis, surveillance, and management of TSC patients [70].

The disorder occurs in approximately 1 in every 6000 to 10,000 individuals, with a population prevalence of approximately 1 in 20,000, impacting both genders and all ethnicities equally. Its wide range of phenotypic expressions can sometimes pose challenges in its recognition. It is estimated that approximately 40,000 Americans and at least 2 million people worldwide are affected by TSC [71].

#### 2.3.1. Molecular Mechanism 

TSC arises from the deletion, reconfiguration, and deactivating mutation of the tumor suppressor genes TSC1 or TSC2, which encode for the proteins hamartin and tuberin, located at loci 9p34 and 16p13, respectively [72]. 

The primary function of these genes is to regulate cellular growth by modulating the phosphatidylinositol 3-kinase signaling pathway, thereby inhibiting the mammalian target of rapamycin (mTOR). The hamartin/tuberin complex plays a crucial role as an inhibitor of tumor development. These proteins curtail the activity of the mTOR pathway, which is responsible for cellular proliferation and the suppression of cellular apoptosis. In individuals with TSC, alterations in these proteins result in sustained activation of the mTOR pathway, leading to the formation of hamartomas in various organs [73]. 

While familial cases of the condition stem from germline mutations and can be heritably transmitted, approximately 70% of TSC patients are affected by somatic mutations, giving rise to sporadic cases. Research indicates that mutations in the TSC2 gene are more prevalent and cause more severe neurological impairments compared to those in TSC1. Familial transmissions often manifest as mild to moderate disease, occasionally not meeting all diagnostic criteria, and are more likely to involve mutations in the TSC1 gene [74].

A “pathogenic” mutation is defined as a mutation that unequivocally hinders protein synthesis and/or disables the function of the TSC1 or TSC2 proteins (e.g., nonsense mutation or frameshift mutations, large genomic deletions), or is a missense mutation whose impact on protein function has been confirmed through functional assessment. Genetic variants in TSC1 and TSC2 with uncertain functional effects are not definitively pathogenic and would not qualify as major diagnostic criteria. A significant fraction (10–25%) of TSC patients do not harbor mutations identified through conventional genetic testing. Therefore, a negative result does not rule out TSC. However, if the mutation present in an affected family member is known, testing for that specific mutation holds considerable predictive value for other family members [75].

The gene products of TSC1 and TSC2 form a functional complex and hinder the phosphorylation of both S6K and 4EBP1, which are critical regulators of translation. Conversely, upregulation of TSC1 and TSC2 suppresses the phosphorylation of S6K and 4EBP1. These findings indicate that one of the primary cellular functions of TSC1/TSC2 is to impede translation by inhibiting the phosphorylation of S6K and 4EBP1. The expression of TSC2 is regulated by cellular energy levels and is pivotal in the cellular energy response pathway. During energy depletion conditions, the AMP-activated protein kinase (AMPK) phosphorylates TSC2, enhancing its activity. AMPK-mediated phosphorylation of TSC2 is crucial for regulating translation and controlling cell size in response to energy scarcity. Additionally, TSC2, and its phosphorylation by AMPK, shields cells from energy deprivation-induced apoptosis [76].

Renal manifestations in TSC pose significant morbidity and mortality risks. Renal complications ranked as the second leading cause of premature death following severe intellectual disability [77]. Angiomyolipomas, benign tumors composed of vascular, smooth muscle, and adipose tissue, are predominantly found in the kidneys of TSC patients, although they can also manifest in other organs. To encompass angiomyolipomas occurring in other sites, the term “angiomyolipomas (N ≥ 2)” was adopted as a key recognized feature, omitting “renal”. Angiomyolipomas are a relatively specific characteristic of TSC, with fat-containing lesions observed in 80% of patients, and fat-poor lesions, although less common, occurring in patients with TSC but rarely in the general population. Angiomyolipomas in the kidneys can lead to significant bleeding due to their vascular nature, potentially necessitating dialysis or even renal transplantation [78]. Multiple renal cysts are infrequently observed in the general populace but may be present in TSC patients harboring mutations in TSC1 or TSC2 or as part of contiguous gene deletion syndromes affecting the TSC2 and PKD1 genes. The TSC2 and PKD1 genes are closely situated and transcribed in opposite directions on chromosome 16p13.3. Deletions encompassing both genes have been documented in a small subset of TSC patients exhibiting both the TSC phenotype and an aggressive PKD phenotype [79].

Examination of murine models of renal cystic disease associated with either Tsc1 or Tsc2 directly or indirectly has revealed that cyst development is linked to particular nephron segments, with all tubular segments implicated in Tsc cyst formation [80,81]. 

Interestingly, in all murine model investigations assessing both mTORC1 activity and targeted cells, there appears to be a discrepancy between the extensive cystic phospho-S6 expression and the notably lower proportion of cells exhibiting Tsc expression loss. One model, in particular, has been associated with a potassium excretion deficiency [81]. Initial research indicated that the majority of renal cysts maintain their Tsc locus integrity, as loss of heterozygosity was detected in only a small subset of cystic epithelial cells [82]. This mirrors the renal cystic disease observed in human TSC cases, where cysts continue to express tuberin and hamartin, differing significantly from the mechanism underlying the formation of angiomyolipomas, which exhibit inactivating mutations and Tsc gene expression loss [83]. A similarly low occurrence of loss of heterozygosity is also observed in autosomal dominant polycystic kidney disease associated with PKD1 mutations, suggesting a potentially unique disease mechanism for such cystic disorders.

In a study by Blissler J.J. et al., a key feature of this mechanism involves a subset of mutant Tsc renal principal epithelial cells or pericytes, which genetically induce or reconfigure normal A-intercalate cells to increase mTORC1 activity, proliferate, and form renal cysts. Similar microenvironmental effects that enhance malignancy involve extracellular vesicles [84]. Extracellular vesicles derived from Tsc1-null cells transform the phenotype of neighboring wild-type cells in vivo, rendering them functionally similar to Tsc1-null cells, a phenomenon sometimes referred to as “phenotypic spreading” [85]. This process of extracellular vesicle initiation is intriguing because as cysts expand the tubular lumen increases in diameter and reduces flow, prolonging the time required for extracellular vesicles to interact with intercalated cells. As cysts enlarge, they detach from tubules, potentially trapping extracellular vesicles in the closed cystic space. Intercalated cells with increased mTORC1 activity are likely to participate in both the loss and uptake of vesicles. H+-ATPase, present in the plasma membranes of various specialized cell types including renal A-intercalate cells, plays a significant role in stimulating chloride secretion through chloride apical channels in the collecting duct. The H+-ATPase interacts amino acid-dependently with the Ragulator complex, recruiting mTORC1 to the lysosomal membrane during amino acid sensing [86]. Genetic deletion of H+-ATPase structural components suppressed amino acid-induced S6K phosphorylation in Drosophila and mammalian cells [87]. Functional inhibition of H+-ATPase activity through chemical inhibitors or mTOR translocation was disrupted by RNAi in lysosomes after amino acid stimulation. These findings indicate that amino acids activate mTORC1 by stimulating its translocation to the lysosomal membrane, where it forms a supercomplex involving H+-ATPase. The activation of mTORC1 may implicate the cystoprotein polycystin-1 (PC-1) in elucidating cystogenesis and could explain the severe phenotype observed in the PKD1/TSC2 contiguous gene syndrome. mTORC1 activity negatively influences the generation of PC-1 and the proper transport of the PC-1/2 complex to cilia. While PC-1 primarily resides on the cilia of principal cells, it is also present on other cell membranes, including intercalated cells, and is highly expressed on extracellular vesicles [88].Genetic studies have shown that mTORC1-mediated PC-1 suppression leads to cyst formation in Tsc1 mutants, potentially clarifying the severe renal manifestations in the PKD1/TSC2 contiguous gene syndrome [89]. Mutations in the TSC2 gene and mutations in the adjacent PKD1 gene lead to the overlapping syndrome of tuberous sclerosis and polycystic kidney disease (Figure 3).

#### 2.3.2. Focus on Kidney Cancer

Renal presentations primarily entail the formation of bilateral multifocal cysts and angiomyolipomas (AMLs: 80%). TSC-associated AMLs may present themselves as bilateral or multifocal growths and exhibit notably accelerated growth rates compared to sporadic cases (1.25 cm/year vs. 0.19 cm/year) [90]. Epithelioid AML, a less common subtype, is recognized as a potentially malignant tumor, with instances of invasion and metastasis reported sporadically. The size of AMLs is directly correlated with the risk of spontaneous hemorrhage, which can occasionally pose life-threatening consequences.

The incidence of RCC in individuals with TSC appears to be only marginally elevated compared to the general population, estimated at approximately 3–5%. However, TSC patients tend to develop malignant tumors at a younger age. Studies on TSC-associated RCCs indicate that the mean age of onset ranges from 30 to 42 years, with a slight predominance observed in females (ratio 2:1) [90].

The spectrum of kidney cancers observed encompasses various histological types, including clear cell, papillary, chromophobe, hybrid tumors, and oncocytomas [91] (Table 1).

The fundamental aspect of radiological diagnosis of angiomyolipomas (AMLs) via non-contrast CT scans is the identification of intra-tumoral fat, characterized by attenuation levels below −10 Hounsfield units (HUs). However, approximately one-third of AMLs associated with tuberous sclerosis complex (TSC) exhibit a paucity of fat and a predominance of muscular tissue, appearing hyperattenuating in comparison to the surrounding parenchyma [92]. Magnetic resonance imaging (MRI) can provide additional diagnostic assistance, as fat-poor AMLs typically display T1 hyperintensity and demonstrate signal loss with fat saturation. Epithelioid AMLs manifest as solid masses on unenhanced scans and appear hypointense on T2-weighted imaging. Guidelines recommend that, following the initial diagnosis, individuals undergo abdominal MRI or CT scans every 2–3 years, starting at childhood, and annual renal function assessment. These scans serve to both diagnose and monitor the growth of angiomyolipomas (AMLs), while also facilitating the early detection of renal cell carcinoma (RCC) [93] (Table 2).

RCC in individuals with TSC frequently exhibits a slow-growing nature, particularly in its early stages. Consequently, a strategy of active monitoring of renal tumors is often practiced until the largest tumor reaches a size of 3 cm.

The size of angiomyolipomas (AMLs) correlates with the risk of hemorrhage; therefore, regular monitoring of AMLs is necessary when they reach a diameter of 4 cm. In such cases, percutaneous selective angio-embolization is performed. 

In cases where renal cell carcinoma (RCC) tumors exceed a diameter of 3 cm, nephron-sparing surgery utilizing enucleation is the favored surgical approach [66]. Given the frequent bilateral, multifocal, and recurrent nature of these tumors, it remains imperative to preserve maximal normal parenchyma to mitigate potential declines in renal function following multiple anticipated surgeries.

Everolimus, an mTOR inhibitor, has been approved for reducing the volume of AMLs in individuals with TSC. It could potentially be used to postpone the necessity of surgery in specific patients tolerating drug side effects [93] according to research conducted by Hatano T. et al. that evaluated 14 patients who received second-line everolimus treatment for TSC-AML after post-transcatheter arterial embolization (TAE), focusing on its efficacy and side effects. Everolimus was given for AMLs up to 4 cm in diameter. The result of AML volume reduction was compared to patients undergoing first-line everolimus therapy. In all patients, AML volume decreased, with a ≥50% reduction in 57% (8 out of 14) of cases, and an average reduction rate of 53%. There was no notable difference in the average reduction rate between second-line and first-line everolimus treatments for TSC-AML post-TAE. Adverse events were minor and aligned with previous findings. In summary, although more research is required, everolimus shows promise as an effective second-line treatment for TSC-AML that recurs after TAE and serves as a feasible therapeutic option [94]. Results of mTOR inhibitor therapy for TSC-associated angiomyolipoma have led to a significant change in the therapeutic algorithm. Previously, embolization was recommended for all lesions larger than 3 cm, but now it is reserved only for cases of acute bleeding. For other patients without contraindications, everolimus therapy is now the primary treatment option. This approach preserves kidney function and prevents renal function loss due to surgical procedures. In TSC patients, this strategy can potentially improve life expectancy, as many have multiple bilateral angiomyolipomas, and a history of surgical interventions, including nephrectomy, is common in adults [95]. The most common side effects included stomatitis, headache, hypercholesterolemia, urinary tract infections, and amenorrhea in female patients. Proteinuria was observed in 15% of patients, mostly classified as grade 1 or 2 [96] (Table 2).

### 2.4. BRCA-1 Associated Protein-1 (BAP-1)-Associated Renal Cell Carcinoma

The hereditary BAP1 tumor syndrome is a relatively uncommon condition characterized by mutations in the BRCA-associated protein-1 gene (BAP1) located on chromosome 3p21.1. It follows an autosomal dominant pattern of inheritance. 

BAP1 syndrome increases the risk of aggressive RCC, cutaneous melanocytic lesions, basal cell carcinoma, mesothelioma (often in the abdomen), and uveal melanoma [97].

#### 2.4.1. Molecular Mechanism

BAP1 belongs to the deubiquitinate (DUB) family of proteins and acts as a ubiquitin carboxy-terminal hydrolase (UCH). It was discovered and named in 1998 as a nuclear protein thought to interact with the RING finger domain of the BRCA1 protein. BAP1 is located on human chromosome 3p21.3, and its encoded protein is present in both the nucleus and the cytoplasm.

Studies investigating the mechanism have shown that BAP1’s tumor suppressor role is associated with its dual function. Within the nucleus, it plays a role in various processes, such as DNA repair and transcription. In the cytoplasm, it governs cell death and mitochondrial metabolism. BAP1 has emerged as a critical tumor suppressor in various cancer types, increasing susceptibility to tumor formation when mutated either in the germline or somatically [98,99].

**Figure 4:**
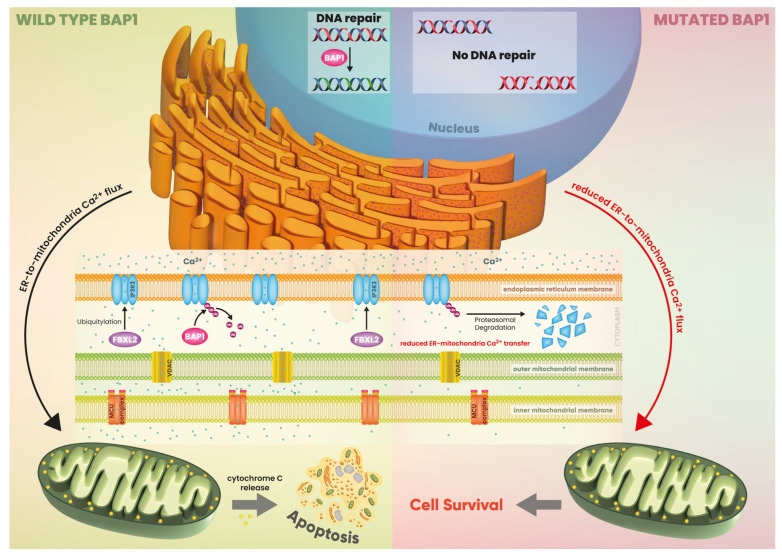
Molecular mechanism of BRCA-1-associated protein-1 (BAP-1)-associated renal cell carcinoma (RCC) [5,6].

Within the cytoplasm, BAP1 localizes to the endoplasmic reticulum (ER), where it interacts with, deubiquitinates, and stabilizes type 3 inositol-1,4,5-triphosphate receptor (IP3R3), thereby regulating ER calcium release and facilitating apoptosis. Decreased BAP1 levels contribute to heightened DNA damage (stemming from reduced nuclear BAP1 activity), diminished apoptosis (attributable to decreased cytoplasmic BAP1 activity), and elevated cellular transformation (resulting from decreased activities in both nuclear and cytoplasmic compartments). Our findings suggest that diminished BAP1 levels promote a tumor phenotype primarily under conditions of genotoxic stress (involving gene–environment interactions). The balance between DNA damage and cell death dictates the outcome: the greater the survival of DNA-damaged cells following exposure, the greater the risk that one of them may progress to malignancy [98] (Figure 4).

#### 2.4.2. Focus on Kidney Cancer

Lesions of RCC related to BAP1 typically display aggressive characteristics, such as a high Fuhrman grade, multifocality, and early onset. Moreover, BAP1-related RCC exhibits the fastest growth rates among all hereditary RCC subtypes, with a rate of 0.6 cm/year. Consequently, active monitoring is not advised for identified lesions [28].

While fewer than 100 families exhibiting this syndrome have been documented, the incidence of RCC is estimated to range from 3% to 10% [100] (Table 1). Somatic loss of BAP1 in sporadic RCC cases has been associated with unfavorable prognostic outcomes [101].

Guidelines have been established for managing individuals with BAP1 syndrome, recommending chest and abdominal imaging every year starting at age 30 (Table 2). Treatment and screening require a collaborative effort involving dermatology, ENT, as well as general and thoracic surgery for potential uveal melanoma and chest/abdominal mesothelioma. Due to the aggressive nature of metastatic disease associated with BAP1 RCC and the limited treatment options available, the “3 cm rule” is not advised. Instead, lesions should be surgically resected with partial nephrectomy, and wide resection should be performed promptly upon lesion discovery [28] (Table 2).

### 2.5. Hereditary Paraganglioma/Pheochromocytoma (HPP) Syndrome/Succinate Dehydrogenase-B Mutation (SDHB)

Recently included in the World Health Organization classification of RCC, succinate dehydrogenase (SDH)-deficient RCC is linked to germline mutations in the genes encoding the SDH subunits (SDHA, SDHB, SDHC, SDHD). Initially identified as a cause of hereditary paraganglioma/pheochromocytoma syndrome, Succinate dehydrogenase (SDH) is a mitochondrial enzyme integral to the Krebs cycle responsible for converting succinate to fumarate.

The age at which RCC typically presents was reported as 30 years, with the youngest documented patient being 14 years old (ranging from 14 to 76 years) [102].

SDH mutations follow an autosomal dominant inheritance pattern, with an estimated incidence of 0.1–0.2% of all renal cancers [103].

A recent review reported that 66% of SDHB mutation carriers had thoraco-abdominal paragangliomas, 25% had head and neck paragangliomas, 12% had pheochromocytomas, 14% had RCC, and 2% had gastrointestinal stromal tumors (GISTs). Patients with SDHB mutation have a relatively high malignancy incidence, ranging from 30% to 41%, with renal cell carcinoma observed in up to 14% and gastrointestinal stromal tumors in 2% of these cases [104].

#### 2.5.1. Molecular Mechanism

Succinate dehydrogenase (SDH), also referred to as mitochondrial respiratory chain complex II, is a crucial enzyme situated on the inner mitochondrial membrane. It serves as a bridge between the tricarboxylic acid (TCA) cycle and oxidative phosphorylation, playing significant roles in both processes. SDH is composed of four subunits encoded by nuclear genes: SDHA, SDHB, SDHC, and SDHD.

Mutations affecting any of these genes deactivate the SDH enzyme, leading to succinate accumulation and increased production of oxygen free radicals. This triggers hypoxia-dependent pathways that further contribute to genetic alterations associated with tumor development [105]. 

Insufficient production of ATP to fulfill the cellular metabolic demands results in an elevated ratio of AMP to ATP. This, in turn, triggers the activation of AMPK, both directly and through phosphorylation at Ser172 of the a-subunit by the tumor suppressor LKB1. AMPK subsequently phosphorylates specific target molecules, such as GSK3 at Ser12 [106]. One outcome of GSK3 phosphorylation is the inhibition of cyclin D1 degradation. Cyclin D1, a member of the cyclin family, propels the G1/S transition in the cell cycle, and its overexpression plays a crucial role in the development of various tumor types [107]. In SDHB-associated RCC, there is AMPK activation, GSK3 phosphorylation, and an accumulation of cyclin D1, suggesting that ATP deficiency and AMPK activation are pivotal in the onset of these renal tumors. Observations further reinforce the notion that AMPK activation in the kidney fosters cell proliferation and survival. Activated AMPK significantly drives mitochondrial biogenesis by activating peroxisome proliferator-activated receptor gamma coactivator-1a (PGC-1a) [108]. 

Mutations in various SDH enzyme subunits are linked to PC-PGL syndromes, characterized by clinical diversity regarding age at onset, tumor site, inheritance pattern, and malignancy risk. The inheritance pattern is autosomal dominant, with penetrance varying widely [109]. 

Mutations in SDHB (located on chromosome 1p36) show the most robust correlation with renal cancer, followed by SDHC. The role of SDHB is pivotal within the tricarboxylic acid (TCA) cycle, exerting varying degrees of influence at different stages of the redox reaction. SDHB serves as an essential constituent of mitochondrial complex II, crucial for the oxidation of succinate to ferredoxin and for facilitating aerobic oxidation and electron transport within the mitochondria. Reduced expression of SDHB promotes aerobic glycolysis, and the absence of SDHB activity is associated with the development of various tumors, including liver and colorectal cancers [110]. 

In kidney cells lacking SDHB, the TCA cycle is fully obstructed, resulting in an enhanced Warburg effect. This effect is even more pronounced in clear cell renal cell carcinoma (ccRCC) cells. The dysfunction of SDHB is strongly correlated with metabolic alterations in kidney cancer cells. Frang et al. demonstrated that SDHB expression was decreased in ccRCC tissues. Elevated methylation of the SDHB gene promoter was responsible for this downregulation. Overexpression of SDHB in ccRCC cells inhibited cell proliferation, colony formation, and migration in vitro, by suppressing glycolysis [109].

One theory posits apoptosis disruption as a consequence of SDH mutations, potentially fostering tumor development by impeding mitochondrial involvement in cell death. Tumor formation stems from the failure to naturally eliminate abnormal cells, allowing them to proliferate unchecked, eventually manifesting malignancy. Abnormal reduction in SDH diminishes damaged cell sensitivity to apoptotic signals, fostering the emergence of new pathways overseen by mutated SDHB, thus yielding abnormal biological repercussions surpassing physiological thresholds [111].

The second theory revolves around oxygen sensing. Loss or mutation of SDHB prompts an abnormal buildup of succinate and reactive oxygen species (ROS) within the body. Elevated succinate levels elevate cancer risk as succinate overflows from mitochondria, inhibiting various enzymes, notably prolyl hydroxylase, which regulates HIF-α. This inhibition leads to HIF-α accumulation, forming a complex with HIF-β that activates downstream genes such as vascular endothelial growth factor (VEGF) and insulin-like growth factor IR (IGFIR). Termed the ‘pseudo-hypoxic mechanism’, this cascade induces new metabolic pathways and vascular pathways in the body. Succinate accumulation also boosts ROS levels, which in turn modulate hypoxia-inducible factors, while impeding effective electron utilization, ultimately inducing a pseudo-hypoxic state in tissue cells [112].

By age 40, only 40% of SDHB mutation carriers develop tumors. The prevalence of disease in asymptomatic carriers screened for the mutation is reported to be less than 22% by age 60. Multifocal disease is less common, with tumors primarily arising from sympathetic ganglia and associated with catecholamine secretion. Head and neck PGLs are less frequent, occurring in about 25% of patients with SDHB mutation [113] (Figure 3).

#### 2.5.2. Focus on Kidney Cancer

SDHx-deficient renal cell carcinoma (RCC) comprises roughly 0.2% of all RCC cases, with 83% of these associated with SDHB subunit germline mutations. Incidence rates are slightly lower in women, with a male-to-female ratio of 1.8:1.0 [114].

The lifetime risk of SDHx-deficient RCC stands at 14%, with up to 26% presenting bilaterally and 33% with metastatic disease, typically with low-grade morphology. Most patients exhibit unilateral kidney tumors, while around 30% experience multifocal or bilateral occurrences [115] (Table 1).

Various subtypes of RCC, such as clear cell, chromophobe, and pRCC type 2 featuring solid and/or cystic components, have been observed in conjunction with these mutations. Despite histological diversity, the consistent hallmark of SDH-deficient RCCs is typically the absence of SDH expression on immunostaining [103]. 

The detection of paragangliomas or pheochromocytomas alongside a renal mass further corroborates the diagnosis of SDH deficiency. Enhanced CT or MRI scans may reveal either unilateral or bilateral renal masses displaying cystic, solid-cystic, or oncocytic characteristics. National guidelines advocate for annual cross-sectional imaging every 2–3 years from childhood for the evaluation of renal lesions. 

For family members at risk of familial pheochromocytomas, paragangliomas, or renal cell carcinoma displaying histopathological features typical of SDH-deficient RCC, it is advisable to undergo germline mutation testing. Due to the potential early onset of paragangliomas, it has been proposed to start genetic testing for at-risk individuals as early as 5 years old. Positive findings necessitate the commencement of annual blood pressure, pulse assessment and annual 24 h urine metanephrines testing for paraganglioma diagnosis from 5 years old onwards [116].

Due to the aggressive nature of SDH-deficient tumors, it is not advisable to pursue active surveillance or ablative techniques. Given the tumors’ aggressive and infiltrative growth patterns, partial nephrectomy with wide margins is preferred. In cases where nephron-sparing surgery with wide margins is not feasible, radical nephrectomy should be considered. Locoregional lymphadenectomy may be warranted for large or complex renal tumors. Currently, there are no FDA-approved systemic therapies specifically tailored for SDH-deficient tumors [66] (Table 2).

### 2.6. Cowden Syndrome

Cowden syndrome (CS), together with Bannayan–Riley–Ruvalcaba syndrome, is included in phosphatase and tensin homolog deleted (PTEN) hamartoma tumor syndrome (PHTS), a condition that mainly predisposes affected individuals to developing hamartomatous growths and oncogenesis in various organ systems.

Cowden syndrome is a hereditary cancer that is often challenging to diagnose. It follows an autosomal dominant inheritance pattern with variable expression, involving a germline mutation in the phosphatase and tensin homolog (PTEN) gene. Approximately 80% of individuals with Cowden syndrome have germline mutations in the PTEN coding sequence [117].

Cowden syndrome is rare, affecting approximately 1 in 200,000 people. Although cancers linked to Cowden syndrome typically develop in adulthood, some of the other symptoms associated with PTEN hamartoma tumor syndrome (PHTS) can be identified in childhood. CS was first recognized as a distinct clinical entity in 1963, nevertheless it was not until 1997 that mutations in PTEN were identified as the cause of CS [118].

Highlighting the significance of PTEN as a tumor suppressor gene, inherited mutations in PTEN have been associated with various autosomal dominant hamartoma syndromes such as Cowden’s disease (OMIM: 308350), Bannayan–Riley–Ruvalcaba syndrome (OMIM: 153480), and Lhermitte–Duclos syndrome (OMIM: 158350). These syndromes are characterized by the presence of benign tumors called hamartomas across multiple organs and an elevated risk of developing various malignancies [119]. 

#### 2.6.1. Molecular Mechanism

The genetic locus associated with Cowden’s disease was mapped in 1996 on chromosome 10q22-23, where the PTEN gene is located. The initial documentation of PTEN mutations in individuals with Cowden syndrome dates back to 1997 [79].

The PTEN gene encodes for a lipid phosphatase, PTEN, which functions as a suppressor of the phosphatidylinositol 3-kinase (PI3K) pathway. Its role involves converting phosphatidylinositol 3,4,5-triphosphate (PIP3) into phosphatidylinositol 4,5-biphosphate [120]. The PTEN gene plays a role in regulating apoptosis and the cell cycle by modulating the phosphatidylinositol 3-kinase (PI3K)/AKT/mammalian target of rapamycin (mTOR) pathways. This intracellular signaling pathway, crucial for cell cycle regulation, is influenced by PTEN activity. The cascade commences with the phosphorylation of PI3K enzymes, resulting in the conversion of phosphatidylinositol to diphosphate (PIP2) and triphosphate (PIP3). PIP3 then triggers AKT activation, which subsequently stimulates mTOR. This mTOR activation is pivotal for processes like protein synthesis, cell growth, proliferation, and inhibition of apoptosis, as depicted in Figure 1. PTEN gene activity modulates the cell cycle by suppressing the PI3K/AKT/mTOR pathway, resulting in reduced cell proliferation and survival, thereby leading to tumorigenesis [121,122].

Individuals who satisfy the clinical standards for CS yet do not exhibit a mutation in the PTEN gene have been identified as possessing germline mutations in SDHB, SDHC, and SDHD. These mutations give rise to “second-hit” mutations, culminating in the heterozygous loss of PTEN. Furthermore, hereditary pheochromocytoma and paraganglioma syndromes stem from these three genetic mutations [119].

Based on research led by J.L. Meister, the incidence of spontaneous PTEN mutation ranges from at least 10.7% to as high as 47.6%. Even if there are no PHTS characteristics, doctors should still consider this diagnosis for patients based on their own relevant medical background [123]. A significantly smaller number of cases are thought to result from mosaicism of PTEN mutations [44].

For individuals with Cowden’s disease, approximately 80% exhibit PTEN germline mutations. These mutations occur in various regions such as the PTEN coding sequence, its promoter, or the 5′ and 3′ untranslated regions. These mutations result in the hindrance of translation or the production of a PTEN protein that is either catalytically inactive, immature, or unstable, possibly leading to rapid degradation [122].

Cowden syndrome (CS) is a complex condition affecting multiple systems, characterized by abnormal tissue growth from all three embryonic origins and heightened susceptibility to thyroid, breast, and potentially other cancers. Additionally, benign lesions in the breast, thyroid, uterus, and skin are prevalent (Table 1). The risk of renal cell carcinoma in individuals with a PTEN mutation was previously undetermined (Figure 3).

#### 2.6.2. Focus on Kidney Cancer

RCC is an underappreciated feature of CS. A recent study from the Cleveland Clinic reported that the lifetime renal cell carcinoma risk may be as high as 34% and age-adjusted standardized incidence ratio (SIR) about 31% [124].

Shuch et al. recently reported that almost 17% (four of 24 patients) of their National Institutes of Health study cohort from 2008 to 2011 with a germline PTEN mutation had a personal history of renal cell carcinoma while none of them had a reported family history of this cancer. Three of these patients were reported to have had solitary renal lesions (two with papillary type 1 and one with clear cell carcinoma) while one presented with bilateral chromophobe renal cell carcinoma (Table 1) [121]. Biannual ultrasound is indicated from 40 years of age. Both of these studies have ascertainment biases, so further research is needed to clarify the incidence of renal cancer in CS (Table 2).

### 2.7. Fumarate Hydratase Tumor Predisposition Syndrome (FHTPS) 

Fumarate hydratase tumor predisposition syndrome (FHTPS) was previously known as hereditary leiomyomatosis and renal cell carcinoma syndrome [125]. Previously identified as Reed’s syndrome, multiple cutaneous and uterine leiomyomatosis (MCUL) (OMIM 150800) is a genetic disorder inherited in an autosomal dominant pattern. When MCUL coincides with various types of renal cancer, it is designated as hereditary leiomyomatosis and renal cell cancer (HLRCC) (OMIM 605839). Both MCUL and HLRCC have been shown to arise from heterozygous germline mutations in the fumarate hydratase (FH) gene, which is thought to act as a tumor suppressor gene under normal conditions.

It is estimated that the population frequency of FHTPS/HLRCC is around 1 in every 200,000 individuals. It is distinguished by the presence of leiomyomas in the skin and uterus. In a few families, pheochromocytoma and paraganglioma have also been reported. Cutaneous leiomyomas typically present as skin-colored to light brown papules or nodules on the trunk and extremities, and occasionally on the face. They generally appear around the age of 30 and increase in size and number with age. Uterine leiomyomas are often numerous and large, with diagnosis occurring between the ages of 18 and 53 [126]. Cutaneous leiomyomas often act as the first signs of MCUL, making dermatologists the primary healthcare providers for these patients (Table 1).

#### 2.7.1. Molecular Mechanism

The genetic locus responsible for MCUL and HLRCC, encoding the FH enzyme, has been associated with chromosome 1q42.3-q43 [127]. The FH enzyme plays a crucial role in converting fumarate to malate within the Krebs cycle. The absence of FH hinders the enzymatic process that converts fumarate to malate. Fumarate can potentially function as an oncometabolite, impeding the function of prolyl hydroxylases responsible for mediating the breakdown of hypoxia-inducible factors. As a result, FH inactivation induces a pseudo-hypoxic state, notably leading to the accumulation of HIF. Increased levels of HIF lead to the activation of numerous oncogenic genes, such as the epidermal growth factor receptor (EGFR), through transcriptional mechanisms [128,129].

Heterozygous germline mutations identified in the FH gene associated with MCUL and HLRCC suggest that these conditions share a genetic basis [130]. Analysis of the FH gene in specific tumors from mantle cell lymphoma (MCL) patients has shown a secondary mutation that deactivates the normal allele, followed by the loss of the wild-type allele. The most researched example is the FH c.1431_1433dupAAA (p.Lys477dup) allele [131]. This pattern is also seen in biopsies of cutaneous and uterine tumors in individuals with MCUL and HLRCC. These observations indicate that FH might act as a tumor suppressor gene. As outlined in a review by N. A. Alam, numerous mutations distributed across the FH gene have been identified thus far. These include missense mutations (58%), frameshift mutations (27%), nonsense mutations (9%), and whole-gene deletions (7%), totaling 73 distinct mutations and continuing to increase in number [132] (Figure 3).

#### 2.7.2. Focus on Kidney Cancer

A portion of individuals affected by MCUL experience the onset with renal cell carcinoma (RCC), accounting for approximately 15.6 percent of FH mutation-positive cases. The average age at which RCC is detected is 44 years. Menko et al. reported that approximately 7% of HLRCC cases were diagnosed before the age of 20, with the earliest case detected at 11 years old [133]. Renal tumors associated with HLRCC usually present as single growths in one kidney. Cases of bilateral RCC in HLRCC are uncommon, with only a few reported instances [134]. The most common form of renal cell carcinoma (RCC) found in HLRCC is type II papillary RCC (PRCC II), followed by collecting duct cancer (CDC). There have also been reported cases of oncocytoma, clear cell carcinoma, and Wilms tumor [135] (Table 1).

PRCC II typically presents enlarged cells with abundant cytoplasm, a notable Fuhrman nuclear grade, and distinctive ‘owl-eye’ nuclei that emphasize thickened elongated papillae when viewed under the microscope.

Most lesions appear heterogeneous with T2-hyperintensity, well-defined margins, and demonstrate limited diffusion on magnetic resonance imaging [136]. Renal lesions can be observed on MRI with poor enhancement, cystic alterations, and local lymphovascular spread, even when the lesion is as small as 1 cm.

In MCUL, about 50% of RCC patients develop metastases, a significantly high rate considering the size and number of primary tumors. The metastasis is attributed as the main cause of mortality in HLRCC (5-year survival up to 30% after tumor detection) [137]. Due to its highly aggressive nature with early documented metastases in tumors as small as 1 cm, active surveillance is not an option, unlike other hereditary tumor syndromes such as VHL and BHD [66,92].

Surgery with nephron-sparing approaches for wide-margin excision represents the cornerstone of treatment as soon as a renal lesion is detected, regardless of lesion size. In cases with large tumors or where wide margins are not feasible, radical nephrectomy is recommended. Retroperitoneal lymph node dissection (i.e., para-aortic and/or entire aorto-caval) should be considered in large or centrally located tumors [66]. Ablation is also not recommended, even for small renal lesions, in HLRCC due to their highly infiltrative biological behavior that may be underestimated on imaging [133].

Several investigations have showcased the notable effectiveness of immunotherapy in individuals with FH-deficient carcinomas, yet contrasting findings have indicated limited advantages from immune checkpoint blockade within this clinical context. A retrospective multicenter study revealed that a combination of multikinase inhibitors with anti-PD1 therapy yielded superior outcomes compared to alternative treatment modalities. The combination of bevacizumab plus erlotinib has shown benefits in some patients with metastatic disease [93].

### 2.8. Hereditary Papillary Renal Cell Carcinoma Syndrome (HPRCC)

Hereditary papillary renal cell carcinoma (HPRCC) is an autosomal dominant disorder marked by the presence of bilateral and multifocal papillary renal cell carcinomas of the classic type.

In 1994, Zbar and colleagues were the first to describe HPRCC in a three-generation family, where 9 out of 15 members exhibited bilateral papillary renal cell tumors. Following this, the same research group reported 41 patients from 10 different families affected by papillary renal cell carcinomas [138,139].

The population incidence of HPRCC is unknown, suggesting that it is a rare condition.

Papillary renal cell carcinoma (pRCC) is the second most common subtype of renal cell carcinoma (RCC) following conventional or clear cell RCC (ccRCC), accounting for approximately 20% of cases. Papillary RCC is further classified into type 1 and type 2 pRCC. Type 1 tumors are linked to MET alterations, whereas type 2 tumors are marked by CDKN2A silencing, SETD2 mutations, TFE3 fusions, and elevated expression of the NRF2-antioxidant response element (ARE) pathway. Although only 13% of sporadic type 1 papillary RCC tumors exhibit MET mutations, alterations in MET occur in 81% of sporadic type 1 papillary tumors due to a combination of missense mutations, gene fusion events, and multiple copies of chromosome 7 (as well as chromosomes 16, 17, and 20). Consequently, MET mutations are relatively uncommon among sporadic type 1 papillary RCC patients [140]. 

In recent decades, significant research has clarified the molecular basis of its tumorigenesis, showing that patients with HPRCC have missense germline mutations in the proto-oncogene MET [141]. In papillary renal carcinomas, mutations occurring in both the germline and somatic forms within the tyrosine kinase domain of the MET proto-oncogene have been observed [141]. The hereditary pattern of HPRC suggests it is inherited dominantly, but not all carriers show symptoms, hinting at incomplete penetrance. Typically, HPRCCs linked to MET arise in adulthood, and nearly everyone with the gene eventually develops the condition [63]. Interestingly, MET mutations or alterations can also occur in various other cancer types such as hepatocellular carcinoma, endometrial cancer, breast cancer, gastric cancer, and squamous cell carcinoma affecting the head and neck region.

#### 2.8.1. Molecular Mechanism

These alterations in MET result in the constant activation of the receptor, a tyrosine kinase found on the surface of renal epithelial cells. Normally, MET plays a role in the repair of renal tubules after events like ischemia, chemical damage, or renal enlargement [141].

The MET gene, situated on chromosome 7 (7q31), holds the blueprint for the transmembrane protein tyrosine kinase, known as c-MET. Its binding partner, hepatocyte growth factor/scatter factor (HGF/SF), is a versatile heparin-binding protein that triggers various cellular responses, including cell motility, proliferation, and resistance to apoptosis, through its mitogenic properties [142].

The pathway involving c-MET and HGF/SF fosters a tumorigenic environment by enhancing cellular motility alongside heightened protease activity. This combination facilitates cellular invasion, allowing cells to breach basement membranes [143].

The defining characteristic of HPRCC’s tumorigenicity lies in the presence of germline proto-oncogene mutations in MET, typically missense mutations. These mutations activate the tyrosine kinase domain of c-MET independently of endogenous HGF/SF. Consequently, this activation initiates downstream signaling pathways that bolster cell survival, proliferation, angiogenesis, and hinder apoptosis [144] (Figure 3).

#### 2.8.2. Focus on Kidney Cancer

Pathogenic alleles of MET are identified in around 0.4% of patients diagnosed with RCC. However, their prevalence significantly increases in individuals with type 1 papillary renal carcinomas, with approximately one out of every eight cases harboring these alleles [145] (Table 1). 

In this syndrome, papillary tumors tend to have lower enhancement, which makes them appear isoechoic and challenging to detect on ultrasound. On a CT scan with IV contrast, these lesions usually show an enhancement of 10–30 HUs [146].

It is noteworthy that amplification of MET copy numbers correlates with cellular dedifferentiation, lymph node invasion, and an elevated risk of metastasis [147]. Small-sized HPRCCs typically exhibit low metastatic potential, hence a “watch-and-wait” approach is commonly adopted for tumors measuring below 3 cm in diameter.

Patients with a family history of MET-activating mutation or those presenting with early onset and multifocal papillary renal cancer undergo germline testing (Table 1). The NCCN advises patients with HPRC to have cross-sectional imaging (CT or MRI) starting at age 30, every 1–2 years, with more frequent surveillance based on the size and/or growth of tumors [93] (Table 2).

Enucleation with minimal normal margins is typically recommended to preserve renal parenchyma.

MET-targeted therapies, such as foretinib, have proven effective in treating metastatic HPRC. However, due to their unfavorable side effect profile, these agents are currently not suitable for use in patients with localized renal tumors [148] (Table 2).

## 3. Conclusions

The discovery of genes linked to inherited renal cancer susceptibility has significantly advanced our understanding of renal tumor pathogenesis. The management strategies for hereditary kidney cancer vary widely among different syndromes in terms of timing and method of intervention. While active surveillance is employed for many syndromes, it is not used for more aggressive ones such as SDH and HLRCC. Similarly, the extent of tumor resection during surgery depends on the tumor’s biological potential. Physicians increasingly recognize patients with hereditary kidney cancer syndromes and refer them for specialist evaluation, resulting in better clinical outcomes with tailored medical management guidelines. The growing availability of high-throughput sequencing is soon expected to identify new RCC-predisposing genes. An effective approach to early diagnosis, surgical removal, and systemic treatment of kidney tumors has significantly increased life expectancy in individuals with RCC-predisposing mutations.

## Figures and Tables

**Table 1:** Gene, extrarenal tumors, incidence, imaging, and histological presentation of hereditary kidney cancer.

Disease	Gene	Extrarenal Tumors	Incidence	Imaging Presentation	Histological Presentation
**Von Hippel–Lindau disease**	VHL	Hemangioblastomas, pancreatic neuroendocrine tumors, pheochromocytomas, paragangliomas	0.3–3.2%	Multiple tumors in both kidneys	Clear cell
**Birt–Hogg–Dubé syndrome**	FLCN	Cystic lung disease, pneumothorax, skin lesions	0.3–1.6%	Bilateral and multifocal renal tumors	50–67% are hybrid oncocytic–chromophobe, 23–34% are chromophobe, 7–9% are clear cell, 3–5% are oncocytomas, and 2% are papillary
**Tuberous sclerosis**	TSC1/TSC2	SEGA (subependymal giant cell astrocytomas, cerebral cortical tubers), heart (rhabdomyomas), lung (lymphangioleiomyomatosis or LAM), and skin (cutaneous and subungual angiofibromas, shagreen patch), retinal hamartomas	0.2–2.6%	Bilateral and multifocal cysts/angiomyolipomas/RCC	Hybrid oncocytic–chromophobe, chromophobe, clear cell, oncocytomas, and papillary
**Succinate dehydrogenase-B** **Mutation (SDHB)/HPP syndrome**	SDHB, SDHC, SDHD, SDHA	PPGLs, thyroid carcinomas, GIST, pituitary adenomas	0.7–0.9%	70% unilateral kidney tumors, 30% multifocal or bilateral	Chromophobe, clear cell, papillary
**Hereditary papillary renal cell carcinoma**	MET	None	0.4%	Multifocal papillary renal cancer	Papillary Renal cell Carcinoma (RCC)
**Fumarate hydratase deficiency**	FH	Uterine leiomyomas	0.2–5.2%	Single growths in one kidney	Papillary, oncocytoma, clear cell RCC
**BAP1 tumor predisposition** **syndrome**	BAP1	Uveal melanomas, malignant mesothelioma	0.3–1.6%	Multifocality	High Fuhrman grade, all RCC subtypes
**Cowden syndrome**	PTEN	Breast carcinomas, thyroid carcinomas, endometrial carcinomas	0.3%	Solitary renal lesions	Papillary and clear cell RCC

**Table 2:** Tumor surveillance, therapy, and surgical approaches of hereditary kidney cancer.

Disease	Tumor Surveillance	Therapy	Surgical Approach
**Von Hippel–Lindau disease**	Biannual TC mdc/MRI from 15 years of age	Anti-HIF2-alpha therapy (belzutifan)	Lesions > 3 cm in diameter
**Birt–Hogg–Dubé syndrome**	Biannual TC mdc/MRI from 20 years of age	/	Lesions > 3 cm in diameter
**Tuberous sclerosis**	TC mdc/MRI every 1–3 years from childhood; annual renal function assessment	mTOR inhibitors (everolimus)	“Watch and wait” for angiomyolipomas and insufficient evidence for RCC
**Succinate dehydrogenase-B** **Mutation (SDHB)/HPP syndrome**	TC mdc/MRI every 2–3 years from childhood	/	Immediate surgical removal
**Hereditary papillary renal cell carcinoma**	Annual TC mdc/MRI from 30 years of age	MET inhibitors	Lesions > 3 cm in diameter
**Fumarate hydratase deficiency**	Annual TC mdc/MRI from 8–10 years of age	Bevacizumab + erlotinib	Immediate surgical removal
**BAP1-tumor predisposition** **syndrome**	Annual TC mdc/MRI from 30 years of age	/	Insufficient evidence, but immediate surgical removal is suggested
**Cowden syndrome**	Biannual ultrasound from 40 years of age	/	Insufficient evidence

## References

[1] Hsieh J.J., Purdue M.P., Signoretti S., Swanton C., Albiges L., Schmidinger M., Heng D.Y., Larkin J., Ficarra V. (2017). Renal Cell Carcinoma. Nat. Rev. Dis. Primers.

[2] Capitanio U., Montorsi F. (2022). Identifying Patients for Adjuvant Therapy after Nephrectomy. Lancet.

[3] Alchoueiry M., Cornejo K., Henske E.P. (2024). Kidney Cancer: Links between Hereditary Syndromes and Sporadic Tumorigenesis. Semin. Diagn. Pathol..

[4] Knudson A.G. (1996). Hereditary Cancer: Two Hits Revisited. J. Cancer Res. Clin. Oncol..

[5] Mighton C., Lerner-Ellis J.P. (2022). Principles of Molecular Testing for Hereditary Cancer. Genes. Chromosomes Cancer.

[6] Imyanitov E.N., Kuligina E.S., Sokolenko A.P., Suspitsin E.N., Yanus G.A., Iyevleva A.G., Ivantsov A.O., Aleksakhina S.N. (2023). Hereditary Cancer Syndromes. World J. Clin. Oncol..

[7] Knudson A.G. (1971). Mutation and Cancer: Statistical Study of Retinoblastoma. Proc. Natl. Acad. Sci. USA.

[8] Latif F., Tory K., Gnarra J., Yao M., Duh F.M., Orcutt M.L., Stackhouse T., Kuzmin I., Modi W., Geil L. (1993). Identification of the von Hippel-Lindau Disease Tumor Suppressor Gene. Science.

[9] Giornale Europeo Di Genetica Umana Prevalenza, Incidenza Alla Nascita e Penetranza Della Malattia Di von Hippel-Lindau (vHL) in Danimarca. https://www.nature.com/articles/ejhg2016173.

[10] Maher E.R., Iselius L., Yates J.R., Littler M., Benjamin C., Harris R., Sampson J., Williams A., Ferguson-Smith M.A., Morton N. (1991). Von Hippel-Lindau Disease: A Genetic Study. J. Med. Genet..

[11] Maddock I.R., Moran A., Maher E.R., Teare M.D., Norman A., Payne S.J., Whitehouse R., Dodd C., Lavin M., Hartley N. (1996). A Genetic Register for von Hippel-Lindau Disease. J. Med. Genet..

[12] Chittiboina P., Lonser R.R. (2015). Von Hippel–Lindau Disease. Handb. Clin. Neurol..

[13] Maher E.R., Neumann H.P., Richard S. (2011). Von Hippel-Lindau Disease: A Clinical and Scientific Review. Eur. J. Hum. Genet..

[14] Knudson A.G. (1986). Genetics of Human Cancer. Annu. Rev. Genet..

[15] Stolle C., Glenn G., Zbar B., Humphrey J.S., Choyke P., Walther M., Pack S., Hurley K., Andrey C., Klausner R. (1998). Improved Detection of Germline Mutations in the von Hippel-Lindau Disease Tumor Suppressor Gene. Hum. Mutat..

[16] Prowse A.H., Webster A.R., Richards F.M., Richard S., Olschwang S., Resche F., Affara N.A., Maher E.R. (1997). Somatic Inactivation of the VHL Gene in Von Hippel-Lindau Disease Tumors. Am. J. Hum. Genet..

[17] Wait S.D., Vortmeyer A.O., Lonser R.R., Chang D.T., Finn M.A., Bhowmick D.A., Pack S.D., Oldfield E.H., Zhuang Z. (2004). Somatic Mutations in VHL Germline Deletion Kindred Correlate with Mild Phenotype. Ann. Neurol..

[18] Friedrich C.A. (1999). Von Hippel-Lindau Syndrome. A Pleomorphic Condition. Cancer.

[19] Iliopoulos O., Ohh M., Kaelin W.G. (1998). pVHL19 Is a Biologically Active Product of the von Hippel-Lindau Gene Arising from Internal Translation Initiation. Proc. Natl. Acad. Sci. USA.

[20] Lee S., Neumann M., Stearman R., Stauber R., Pause A., Pavlakis G.N., Klausner R.D. (1999). Transcription-Dependent Nuclear-Cytoplasmic Trafficking Is Required for the Function of the von Hippel-Lindau Tumor Suppressor Protein. Mol. Cell Biol..

[21] Lee S., Chen D.Y., Humphrey J.S., Gnarra J.R., Linehan W.M., Klausner R.D. (1996). Nuclear/Cytoplasmic Localization of the von Hippel-Lindau Tumor Suppressor Gene Product Is Determined by Cell Density. Proc. Natl. Acad. Sci. USA.

[22] Li M., Kim W.Y. (2011). Two Sides to Every Story: The HIF-Dependent and HIF-Independent Functions of pVHL. J. Cell Mol. Med..

[23] Wang V., Davis D.A., Haque M., Huang L.E., Yarchoan R. (2005). Differential Gene Up-Regulation by Hypoxia-Inducible Factor-1alpha and Hypoxia-Inducible Factor-2alpha in HEK293T Cells. Cancer Res..

[24] Mandriota S.J., Turner K.J., Davies D.R., Murray P.G., Morgan N.V., Sowter H.M., Wykoff C.C., Maher E.R., Harris A.L., Ratcliffe P.J. (2002). HIF Activation Identifies Early Lesions in VHL Kidneys: Evidence for Site-Specific Tumor Suppressor Function in the Nephron. Cancer Cell.

[25] Kaelin W.G. (2002). Molecular Basis of the VHL Hereditary Cancer Syndrome. Nat. Rev. Cancer.

[26] Crossey P.A., Eng C., Ginalska-Malinowska M., Lennard T.W., Wheeler D.C., Ponder B.A., Maher E.R. (1995). Molecular Genetic Diagnosis of von Hippel-Lindau Disease in Familial Phaeochromocytoma. J. Med. Genet..

[27] PubMed Coinvolgimento Renale Nella Malattia Di von Hippel-Lindau. https://pubmed.ncbi.nlm.nih.gov/8872970/.

[28] Ball M.W., An J.Y., Gomella P.T., Gautam R., Ricketts C.J., Vocke C.D., Schmidt L.S., Merino M.J., Srinivasan R., Malayeri A.A. (2020). Growth Rates of Genetically Defined Renal Tumors: Implications for Active Surveillance and Intervention. J. Clin. Oncol..

[29] Nielsen S.M., Rhodes L., Blanco I., Chung W.K., Eng C., Maher E.R., Richard S., Giles R.H. (2016). Von Hippel-Lindau Disease: Genetics and Role of Genetic Counseling in a Multiple Neoplasia Syndrome. J. Clin. Oncol..

[30] Steinbach F., Novick A.C., Zincke H., Miller D.P., Williams R.D., Lund G., Skinner D.G., Esrig D., Richie J.P., deKernion J.B. (1995). Treatment of Renal Cell Carcinoma in von Hippel-Lindau Disease: A Multicenter Study. J. Urol..

[31] Duffey B.G., Choyke P.L., Glenn G., Grubb R.L., Venzon D., Linehan W.M., Walther M.M. (2004). The Relationship between Renal Tumor Size and Metastases in Patients with von Hippel-Lindau Disease. J. Urol..

[32] Iacovelli R., Arduini D., Ciccarese C., Pierconti F., Strusi A., Piro G., Carbone C., Foschi N., Daniele G., Tortora G. (2022). Targeting Hypoxia-Inducible Factor Pathways in Sporadic and Von Hippel-Lindau Syndrome-Related Kidney Cancers. Crit. Rev. Oncol. Hematol..

[33] Cho H., Du X., Rizzi J.P., Liberzon E., Chakraborty A.A., Gao W., Carvo I., Signoretti S., Bruick R.K., Josey J.A. (2016). On-Target Efficacy of a HIF-2α Antagonist in Preclinical Kidney Cancer Models. Nature.

[34] Muller M.-E., Daccord C., Taffé P., Lazor R. (2021). Prevalence of Birt-Hogg-Dubé Syndrome Determined through Epidemiological Data on Spontaneous Pneumothorax and Bayes Theorem. Front. Med. (Lausanne).

[35] Yang Y., Padilla-Nash H.M., Vira M.A., Abu-Asab M.S., Val D., Worrell R., Tsokos M., Merino M.J., Pavlovich C.P., Ried T. (2008). The UOK 257 Cell Line—A Novel Model for Studies of the Human Birt-Hogg-Dubé Gene Pathway. Cancer Genet. Cytogenet..

[36] Possik E., Jalali Z., Nouët Y., Yan M., Gingras M.-C., Schmeisser K., Panaite L., Dupuy F., Kharitidi D., Chotard L. (2014). Folliculin Regulates Ampk-Dependent Autophagy and Metabolic Stress Survival. PLoS Genet..

[37] Hasumi Y., Baba M., Ajima R., Hasumi H., Valera V.A., Klein M.E., Haines D.C., Merino M.J., Hong S.-B., Yamaguchi T.P. (2009). Homozygous Loss of BHD Causes Early Embryonic Lethality and Kidney Tumor Development with Activation of mTORC1 and mTORC2. Proc. Natl. Acad. Sci. USA.

[38] Tsun Z.-Y., Bar-Peled L., Chantranupong L., Zoncu R., Wang T., Kim C., Spooner E., Sabatini D.M. (2013). The Folliculin Tumor Suppressor Is a GAP for the RagC/D GTPases That Signal Amino Acid Levels to mTORC1. Mol. Cell.

[39] PubMed Ruolo Protettivo dell’autofagia Contro la Citolisina di Vibrio Cholerae, una Tossina Formante Pori Di V. Cholerae. https://pubmed.ncbi.nlm.nih.gov/17267617/.

[40] Shintani T., Klionsky D.J. (2004). Cargo Proteins Facilitate the Formation of Transport Vesicles in the Cytoplasm to Vacuole Targeting Pathway. J. Biol. Chem..

[41] Egan D.F., Shackelford D.B., Mihaylova M.M., Gelino S., Kohnz R.A., Vasquez D.A., Joshi A., Gwinn D.M., Taylor R., Asara J.M. (2011). Phosphorylation of ULK1 (hATG1) by AMP-Activated Protein Kinase Connects Energy Sensing to Mitophagy. Science.

[42] Djeddi A., Michelet X., Culetto E., Alberti A., Barois N., Legouis R. (2012). Induction of Autophagy in ESCRT Mutants Is an Adaptive Response for Cell Survival in C. Elegans. J. Cell Sci..

[43] Samokhvalov V., Scott B.A., Crowder C.M. (2008). Autophagy Protects from Hypoxic Injury in C. Elegans. Autophagy.

[44] Betschinger J., Nichols J., Dietmann S., Corrin P.D., Paddison P.J., Smith A. (2013). Exit from Pluripotency Is Gated by Intracellular Redistribution of the bHLH Transcription Factor Tfe3. Cell.

[45] Mathieu J., Detraux D., Kuppers D., Wang Y., Cavanaugh C., Sidhu S., Levy S., Robitaille A.M., Ferreccio A., Bottorff T. (2019). Folliculin Regulates mTORC1/2 and WNT Pathways in Early Human Pluripotency. Nat. Commun..

[46] Di Malta C., Siciliano D., Calcagni A., Monfregola J., Punzi S., Pastore N., Eastes A.N., Davis O., De Cegli R., Zampelli A. (2017). Transcriptional Activation of RagD GTPase Controls mTORC1 and Promotes Cancer Growth. Science.

[47] Dunlop E.A., Seifan S., Claessens T., Behrends C., Kamps M.A., Rozycka E., Kemp A.J., Nookala R.K., Blenis J., Coull B.J. (2014). FLCN, a Novel Autophagy Component, Interacts with GABARAP and Is Regulated by ULK1 Phosphorylation. Autophagy.

[48] PMC I Tumori Renali di Birt-Hogg-Dubé Sono Geneticamente Distinti dalle Altre Neoplasie Renali e Sono Associati ad una Sovraregolazione dell’Espressione Genica Mitocondriale. https://www.ncbi.nlm.nih.gov/pmc/articles/PMC3012009/.

[49] Luijten M.N.H., Basten S.G., Claessens T., Vernooij M., Scott C.L., Janssen R., Easton J.A., Kamps M.A.F., Vreeburg M., Broers J.L.V. (2013). Birt-Hogg-Dube Syndrome Is a Novel Ciliopathy. Hum. Mol. Genet..

[50] Glykofridis I.E., Knol J.C., Balk J.A., Westland D., Pham T.V., Piersma S.R., Lougheed S.M., Derakhshan S., Veen P., Rooimans M.A. (2021). Loss of FLCN-FNIP1/2 Induces a Non-Canonical Interferon Response in Human Renal Tubular Epithelial Cells. eLife.

[51] El-Houjeiri L., Possik E., Vijayaraghavan T., Paquette M., Martina J.A., Kazan J.M., Ma E.H., Jones R., Blanchette P., Puertollano R. (2019). The Transcription Factors TFEB and TFE3 Link the FLCN-AMPK Signaling Axis to Innate Immune Response and Pathogen Resistance. Cell Rep..

[52] Endoh M., Baba M., Endoh T., Hirayama A., Nakamura-Ishizu A., Umemoto T., Hashimoto M., Nagashima K., Soga T., Lang M. (2020). A FLCN-TFE3 Feedback Loop Prevents Excessive Glycogenesis and Phagocyte Activation by Regulating Lysosome Activity. Cell Rep..

[53] Napolitano G., Di Malta C., Esposito A., de Araujo M.E.G., Pece S., Bertalot G., Matarese M., Benedetti V., Zampelli A., Stasyk T. (2020). A Substrate-Specific mTORC1 Pathway Underlies Birt–Hogg–Dubé Syndrome. Nature.

[54] Taya M., Hammes S.R. (2018). Glycoprotein Non-Metastatic Melanoma Protein B (GPNMB) and Cancer: A Novel Potential Therapeutic Target. Steroids.

[55] Rose A.A.N., Biondini M., Curiel R., Siegel P.M. (2017). Targeting GPNMB with Glembatumumab Vedotin: Current Developments and Future Opportunities for the Treatment of Cancer. Pharmacol. Ther..

[56] Näf E., Laubscher D., Hopfer H., Streit M., Matyas G. (2016). Birt–Hogg–Dubé Syndrome: Novel FLCN Frameshift Deletion in Daughter and Father with Renal Cell Carcinomas. Fam. Cancer.

[57] Pavlovich C.P., Grubb R.L., Hurley K., Glenn G.M., Toro J., Schmidt L.S., Torres-Cabala C., Merino M.J., Zbar B., Choyke P. (2005). Evaluation and Management of Renal Tumors in the Birt-Hogg-Dubé Syndrome. J. Urol..

[58] Data from: Renal Imaging in 199 Dutch Patients with Birt-Hogg-Dubé Syndrome: Screening Compliance and Outcome. https://researchinformation.amsterdamumc.org/en/datasets/data-from-renal-imaging-in-199-dutch-patients-with-birt-hogg-dub%C3%A9.

[59] Vocke C.D., Yang Y., Pavlovich C.P., Schmidt L.S., Nickerson M.L., Torres-Cabala C.A., Merino M.J., Walther M.M., Zbar B., Linehan W.M. (2005). High Frequency of Somatic Frameshift BHD Gene Mutations in Birt-Hogg-Dubé-Associated Renal Tumors. J. Natl. Cancer Inst..

[60] Castellucci R., Marchioni M., Valenti S., Sortino G., Borgonovo G., Pesenti N., Vismara A.C.A., Circo M.C., Sessa B., Micheli E. (2017). Multiple Chromophobe and Clear Cell Renal Cancer in a Patient Affected by Birt-Hogg-Dubè Syndrome: A Case Report. Urologia.

[61] Linehan W.M. (2013). Evaluation and Screening for Hereditary Renal Cell Cancers. Can. Urol. Assoc. J..

[62] Ball M.W., Srinivasan R. (2018). Kidney Cancer in 2017: Challenging and Refining Treatment Paradigms. Nat. Rev. Urol..

[63] Carlo M.I., Hakimi A.A., Stewart G.D., Bratslavsky G., Brugarolas J., Chen Y.-B., Linehan W.M., Maher E.R., Merino M.J., Offit K. (2019). Familial Kidney Cancer: Implications of New Syndromes and Molecular Insights. Eur. Urol..

[64] Rosenkrantz A.B., Hindman N., Fitzgerald E.F., Niver B.E., Melamed J., Babb J.S. (2010). MRI Features of Renal Oncocytoma and Chromophobe Renal Cell Carcinoma. AJR Am. J. Roentgenol..

[65] Bratslavsky G., Mendhiratta N., Daneshvar M., Brugarolas J., Ball M.W., Metwalli A., Nathanson K.L., Pierorazio P.M., Boris R.S., Singer E.A. (2021). Genetic Risk Assessment for Hereditary Renal Cell Carcinoma: Clinical Consensus Statement. Cancer.

[66] Gomella P.T., Linehan W.M., Ball M.W. (2021). Precision Surgery and Kidney Cancer: Knowledge of Genetic Alterations Influences Surgical Management. Genes.

[67] Stamatakis L., Metwalli A.R., Middelton L.A., Marston Linehan W. (2013). Diagnosis and Management of BHD-Associated Kidney Cancer. Fam. Cancer.

[68] Nakamura M., Yao M., Sano F., Sakata R., Tatenuma T., Makiyama K., Nakaigawa N., Kubota Y. (2013). A case of metastatic renal cell carcinoma associated with Birt-Hogg-Dubé syndrome treated with molecular-targeting agents. Hinyokika Kiyo.

[69] Hinton R.B., Prakash A., Romp R.L., Krueger D.A., Knilans T.K., International Tuberous Sclerosis Consensus Group (2014). Cardiovascular Manifestations of Tuberous Sclerosis Complex and Summary of the Revised Diagnostic Criteria and Surveillance and Management Recommendations from the International Tuberous Sclerosis Consensus Group. J. Am. Heart Assoc..

[70] Northrup H., Krueger D.A., International Tuberous Sclerosis Complex Consensus Group (2013). Tuberous Sclerosis Complex Diagnostic Criteria Update: Recommendations of the 2012 Iinternational Tuberous Sclerosis Complex Consensus Conference. Pediatr. Neurol..

[71] DiMario F.J., Sahin M., Ebrahimi-Fakhari D. (2015). Tuberous Sclerosis Complex. Pediatr. Clin. N. Am..

[72] Sadowski K., Kotulska K., Schwartz R.A., Jóźwiak S. (2016). Systemic Effects of Treatment with mTOR Inhibitors in Tuberous Sclerosis Complex: A Comprehensive Review. J. Eur. Acad. Dermatol. Venereol..

[73] MacKeigan J.P., Krueger D.A. (2015). Differentiating the mTOR Inhibitors Everolimus and Sirolimus in the Treatment of Tuberous Sclerosis Complex. Neuro Oncol..

[74] Ng K.H., Ng S.M., Parker A. (2015). Annual Review of Children with Tuberous Sclerosis. Arch. Dis. Child.-Educ. Pract..

[75] Hoogeveen-Westerveld M., Ekong R., Povey S., Mayer K., Lannoy N., Elmslie F., Bebin M., Dies K., Thompson C., Sparagana S.P. (2013). Functional Assessment of TSC2 Variants Identified in Individuals with Tuberous Sclerosis Complex. Hum. Mutat..

[76] Inoki K., Zhu T., Guan K.-L. (2003). TSC2 Mediates Cellular Energy Response to Control Cell Growth and Survival. Cell.

[77] PMC Renal Manifestations of Tuberous Sclerosis Complex. https://www.ncbi.nlm.nih.gov/pmc/articles/PMC7478169/.

[78] Bissler J.J., Kingswood J.C. (2004). Renal Angiomyolipomata. Kidney Int..

[79] Brook-Carter P.T., Peral B., Ward C.J., Thompson P., Hughes J., Maheshwar M.M., Nellist M., Gamble V., Harris P.C., Sampson J.R. (1994). Deletion of the TSC2 and PKD1 Genes Associated with Severe Infantile Polycystic Kidney Disease—A Contiguous Gene Syndrome. Nat. Genet..

[80] Armour E.A., Carson R.P., Ess K.C. (2012). Cystogenesis and Elongated Primary Cilia in Tsc1-Deficient Distal Convoluted Tubules. Am. J. Physiol. Ren. Physiol..

[81] Chen Z., Dong H., Jia C., Song Q., Chen J., Zhang Y., Lai P., Fan X., Zhou X., Liu M. (2014). Activation of mTORC1 in Collecting Ducts Causes Hyperkalemia. J. Am. Soc. Nephrol..

[82] Wilson C., Bonnet C., Guy C., Idziaszczyk S., Colley J., Humphreys V., Maynard J., Sampson J.R., Cheadle J.P. (2006). *Tsc1* Haploinsufficiency without Mammalian Target of Rapamycin Activation Is Sufficient for Renal Cyst Formation in *Tsc1^+/−^* Mice. Cancer Res..

[83] Bonsib S.M., Boils C., Gokden N., Grignon D., Gu X., Higgins J.P.T., Leroy X., McKenney J.K., Nasr S.H., Phillips C. (2016). Tuberous Sclerosis Complex: Hamartin and Tuberin Expression in Renal Cysts and Its Discordant Expression in Renal Neoplasms. Pathol. Res. Pract..

[84] Zomer A., Maynard C., Verweij F.J., Kamermans A., Schäfer R., Beerling E., Schiffelers R.M., de Wit E., Berenguer J., Ellenbroek S.I.J. (2015). In Vivo Imaging Reveals Extracellular Vesicle-Mediated Phenocopying of Metastatic Behavior. Cell.

[85] Patel B., Patel J., Cho J.-H., Manne S., Bonala S., Henske E., Roegiers F., Markiewski M., Karbowniczek M. (2016). Exosomes Mediate the Acquisition of the Disease Phenotypes by Cells with Normal Genome in Tuberous Sclerosis Complex. Oncogene.

[86] Kim J., Kim E. (2016). Rag GTPase in Amino Acid Signaling. Amino Acids.

[87] Zoncu R., Bar-Peled L., Efeyan A., Wang S., Sancak Y., Sabatini D.M. (2011). mTORC1 Senses Lysosomal Amino Acids through an Inside-out Mechanism That Requires the Vacuolar H^+^-ATPase. Science.

[88] Kim H., Xu H., Yao Q., Li W., Huang Q., Outeda P., Cebotaru V., Chiaravalli M., Boletta A., Piontek K. (2014). Ciliary Membrane Proteins Traffic through the Golgi via a Rabep1/GGA1/Arl3-Dependent Mechanism. Nat. Commun..

[89] Pema M., Drusian L., Chiaravalli M., Castelli M., Yao Q., Ricciardi S., Somlo S., Qian F., Biffo S., Boletta A. (2016). mTORC1-Mediated Inhibition of Polycystin-1 Expression Drives Renal Cyst Formation in Tuberous Sclerosis Complex. Nat. Commun..

[90] Anglickis M., Anglickienė G., Andreikaitė G., Skrebūnas A. (2019). Microwave Thermal Ablation versus Open Partial Nephrectomy for the Treatment of Small Renal Tumors in Patients Over 70 Years Old. Medicina.

[91] Gupta S., Jimenez R.E., Herrera-Hernandez L., Lohse C.M., Thompson R.H., Boorjian S.A., Leibovich B.C., Cheville J.C. (2021). Renal Neoplasia in Tuberous Sclerosis: A Study of 41 Patients. Mayo Clin. Proc..

[92] Gaur S., Turkbey B., Choyke P. (2017). Hereditary Renal Tumor Syndromes: Update on Diagnosis and Management. Semin. Ultrasound CT MRI.

[93] Motzer R.J., Jonasch E., Agarwal N., Alva A., Baine M., Beckermann K., Carlo M.I., Choueiri T.K., Costello B.A., Derweesh I.H. (2022). Kidney Cancer, Version 3.2022, NCCN Clinical Practice Guidelines in Oncology. J. Natl. Compr. Cancer Netw..

[94] Hatano T., Matsu-Ura T., Mori K.-I., Inaba H., Endo K., Tamari M., Egawa S. (2018). Effect of Everolimus Treatment for Regrown Renal Angiomyolipoma Associated with Tuberous Sclerosis Complex after Transcatheter Arterial Embolization. Int. J. Clin. Oncol..

[95] Pirson Y. (2013). Tuberous Sclerosis Complex-Associated Kidney Angiomyolipoma: From Contemplation to Action. Nephrol. Dial. Transpl..

[96] Bissler J., Cappell K., Charles H., Song X., Liu Z., Prestifilippo J., Hulbert J. (2015). Rates of Interventional Procedures in Patients with Tuberous Sclerosis Complex-Related Renal Angiomyolipoma. Curr. Med. Res. Opin..

[97] Testa J.R., Cheung M., Pei J., Below J.E., Tan Y., Sementino E., Cox N.J., Dogan A.U., Pass H.I., Trusa S. (2011). Germline BAP1 Mutations Predispose to Malignant Mesothelioma. Nat. Genet..

[98] PubMed BAP1 Regola Il Flusso di Ca^2+^ Mediato da IP3R3 Verso i Mitocondri Sopprimendo la Trasformazione Cellulare. https://pubmed.ncbi.nlm.nih.gov/28614305/.

[99] Peña-Llopis S., Vega-Rubín-de-Celis S., Liao A., Leng N., Pavía-Jiménez A., Wang S., Yamasaki T., Zhrebker L., Sivanand S., Spence P. (2012). BAP1 Loss Defines a New Class of Renal Cell Carcinoma. Nat. Genet..

[100] Rai K., Pilarski R., Cebulla C.M., Abdel-Rahman M.H. (2016). Comprehensive Review of BAP1 Tumor Predisposition Syndrome with Report of Two New Cases. Clin. Genet..

[101] Slane B.G., Aykin-Burns N., Smith B.J., Kalen A.L., Goswami P.C., Domann F.E., Spitz D.R. (2006). Mutation of Succinate Dehydrogenase Subunit C Results in Increased O_2_^.−^, Oxidative Stress, and Genomic Instability. Cancer Res..

[102] Ricketts C.J., Shuch B., Vocke C.D., Metwalli A.R., Bratslavsky G., Middelton L., Yang Y., Wei M.-H., Pautler S.E., Peterson J. (2012). Succinate Dehydrogenase Kidney Cancer: An Aggressive Example of the Warburg Effect in Cancer. J. Urol..

[103] Gill A.J., Hes O., Papathomas T., Šedivcová M., Tan P.H., Agaimy A., Andresen P.A., Kedziora A., Clarkson A., Toon C.W. (2014). Succinate Dehydrogenase (SDH)-Deficient Renal Carcinoma: A Morphologically Distinct Entity: A Clinicopathologic Series of 36 Tumors from 27 Patients. Am. J. Surg. Pathol..

[104] Benn D.E., Robinson B.G., Clifton-Bligh R.J. (2015). 15 YEARS OF PARAGANGLIOMA: Clinical Manifestations of Paraganglioma Syndromes Types 1–5. Endocr. Relat. Cancer.

[105] Benn D.E., Gimenez-Roqueplo A.-P., Reilly J.R., Bertherat J., Burgess J., Byth K., Croxson M., Dahia P.L.M., Elston M., Gimm O. (2006). Clinical Presentation and Penetrance of Pheochromocytoma/Paraganglioma Syndromes. J. Clin. Endocrinol. Metab..

[106] Hardie D.G. (2011). AMP-Activated Protein Kinase: A Cellular Energy Sensor with a Key Role in Metabolic Disorders and in Cancer. Biochem. Soc. Trans..

[107] Alao J.P., Stavropoulou A.V., Lam E.W.-F., Coombes R.C. (2006). Role of Glycogen Synthase Kinase 3 Beta (GSK3β) in Mediating the Cytotoxic Effects of the Histone Deacetylase Inhibitor Trichostatin A (TSA) in MCF-7 Breast Cancer Cells. Mol. Cancer.

[108] Hardie D.G., Ross F.A., Hawley S.A. (2012). AMPK: A Nutrient and Energy Sensor That Maintains Energy Homeostasis. Nat. Rev. Mol. Cell Biol..

[109] Ricketts C.J., Forman J.R., Rattenberry E., Bradshaw N., Lalloo F., Izatt L., Cole T.R., Armstrong R., Kumar V.K.A., Morrison P.J. (2010). Tumor Risks and Genotype-Phenotype-Proteotype Analysis in 358 Patients with Germline Mutations in SDHB and SDHD. Hum. Mutat..

[110] Tomitsuka E., Hirawake H., Goto Y., Taniwaki M., Harada S., Kita K. (2003). Direct Evidence for Two Distinct Forms of the Flavoprotein Subunit of Human Mitochondrial Complex II (Succinate-Ubiquinone Reductase). J. Biochem..

[111] Bayley J.-P., Devilee P., Taschner P.E.M. (2005). The SDH Mutation Database: An Online Resource for Succinate Dehydrogenase Sequence Variants Involved in Pheochromocytoma, Paraganglioma and Mitochondrial Complex II Deficiency. BMC Med. Genet..

[112] Sun F., Huo X., Zhai Y., Wang A., Xu J., Su D., Bartlam M., Rao Z. (2005). Crystal Structure of Mitochondrial Respiratory Membrane Protein Complex II. Cell.

[113] Timmers H.J.L.M., Kozupa A., Eisenhofer G., Raygada M., Adams K.T., Solis D., Lenders J.W.M., Pacak K. (2007). Clinical Presentations, Biochemical Phenotypes, and Genotype-Phenotype Correlations in Patients with Succinate Dehydrogenase Subunit B-Associated Pheochromocytomas and Paragangliomas. J. Clin. Endocrinol. Metab..

[114] Kuroda N., Yorita K., Nagasaki M., Harada Y., Ohe C., Jeruc J., Raspollini M.R., Michal M., Hes O., Amin M.B. (2016). Review of Succinate Dehydrogenase-Deficient Renal Cell Carcinoma with Focus on Clinical and Pathobiological Aspects. Pol. J. Pathol..

[115] Williamson S.R., Eble J.N., Amin M.B., Gupta N.S., Smith S.C., Sholl L.M., Montironi R., Hirsch M.S., Hornick J.L. (2015). Succinate Dehydrogenase-Deficient Renal Cell Carcinoma: Detailed Characterization of 11 Tumors Defining a Unique Subtype of Renal Cell Carcinoma. Mod. Pathol..

[116] Tufton N., Shapiro L., Sahdev A., Kumar A.V., Martin L., Drake W.M., Akker S.A., Storr H.L. (2019). An Analysis of Surveillance Screening for SDHB-Related Disease in Childhood and Adolescence. Endocr. Connect..

[117] Pilarski R. (2009). Cowden Syndrome: A Critical Review of the Clinical Literature. J. Genet. Couns..

[118] Lloyd K.M., Dennis M. (1963). Cowden’s Disease. A Possible New Symptom Complex with Multiple System Involvement. Ann. Intern. Med..

[119] Dragoo D.D., Taher A., Wong V.K., Elsaiey A., Consul N., Mahmoud H.S., Mujtaba B., Stanietzky N., Elsayes K.M. (2021). PTEN Hamartoma Tumor Syndrome/Cowden Syndrome: Genomics, Oncogenesis, and Imaging Review for Associated Lesions and Malignancy. Cancers.

[120] Maehama T., Dixon J.E. (1998). The Tumor Suppressor, PTEN/MMAC1, Dephosphorylates the Lipid Second Messenger, Phosphatidylinositol 3,4,5-Trisphosphate. J. Biol. Chem..

[121] Gammon A., Jasperson K., Champine M. (2016). Genetic Basis of Cowden Syndrome and Its Implications for Clinical Practice and Risk Management. Appl. Clin. Genet..

[122] Squarize C.H., Castilho R.M., Gutkind J.S. (2008). Chemoprevention and Treatment of Experimental Cowden’s Disease by mTOR Inhibition with Rapamycin. Cancer Res..

[123] Mester J.L., Eng C. (2012). Estimate of de Novo Mutation Frequency in Probands with PTEN Hamartoma Tumor Syndrome. Genet. Med..

[124] Tan M.-H., Mester J.L., Ngeow J., Rybicki L.A., Orloff M.S., Eng C. (2012). Lifetime Cancer Risks in Individuals with Germline PTEN Mutations. Clin. Cancer Res..

[125] Carlo M.I. (2023). Hereditary Renal Cell Carcinoma Syndromes. Hematol./Oncol. Clin. N. Am..

[126] Kamihara J., Schultz K.A., Rana H.Q., Adam M.P., Feldman J., Mirzaa G.M., Pagon R.A., Wallace S.E., Bean L.J., Gripp K.W., Amemiya A. (1993). FH Tumor Predisposition Syndrome. GeneReviews^®^.

[127] Alam N.A., Bevan S., Churchman M., Barclay E., Barker K., Jaeger E.E., Nelson H.M., Healy E., Pembroke A.C., Friedmann P.S. (2001). Localization of a Gene (MCUL1) for Multiple Cutaneous Leiomyomata and Uterine Fibroids to Chromosome 1q42.3-Q43. Am. J. Hum. Genet..

[128] Sulkowski P.L., Sundaram R.K., Oeck S., Corso C.D., Liu Y., Noorbakhsh S., Niger M., Boeke M., Ueno D., Kalathil A.N. (2018). Krebs-Cycle-Deficient Hereditary Cancer Syndromes Are Defined by Defects in Homologous-Recombination DNA Repair. Nat. Genet..

[129] Ricketts C.J., Killian J.K., Vocke C.D., Wang Y., Merino M.J., Meltzer P.S., Linehan W.M. (2022). Kidney Tumors Associated with Germline Mutations of FH and SDHB Show a CpG Island Methylator Phenotype (CIMP). PLoS ONE.

[130] Toro J.R., Glenn G., Hou L., Duray P., Clark B., Merino M., Zbar B., Linehan M., Turner M.L. (2003). Facial Papules, Spontaneous Pneumothorax, and Renal Tumors. J. Am. Acad. Dermatol..

[131] Zhang L., Walsh M.F., Jairam S., Mandelker D., Zhong Y., Kemel Y., Chen Y.-B., Musheyev D., Zehir A., Jayakumaran G. (2020). Fumarate Hydratase FH c.1431_1433dupAAA (p.Lys477dup) Variant Is Not Associated with Cancer Including Renal Cell Carcinoma. Hum. Mutat..

[132] Alam N.A., Olpin S., Leigh I.M. (2005). Fumarate Hydratase Mutations and Predisposition to Cutaneous Leiomyomas, Uterine Leiomyomas and Renal Cancer. Br. J. Dermatol..

[133] Menko F.H., Maher E.R., Schmidt L.S., Middelton L.A., Aittomäki K., Tomlinson I., Richard S., Linehan W.M. (2014). Hereditary Leiomyomatosis and Renal Cell Cancer (HLRCC): Renal Cancer Risk, Surveillance and Treatment. Fam. Cancer.

[134] Lehtonen H.J., Kiuru M., Ylisaukko-Oja S.K., Salovaara R., Herva R., Koivisto P.A., Vierimaa O., Aittomäki K., Pukkala E., Launonen V. (2006). Increased Risk of Cancer in Patients with Fumarate Hydratase Germline Mutation. J. Med. Genet..

[135] Merino M.J., Torres-Cabala C., Pinto P., Linehan W.M. (2007). The Morphologic Spectrum of Kidney Tumors in Hereditary Leiomyomatosis and Renal Cell Carcinoma (HLRCC) Syndrome. Am. J. Surg. Pathol..

[136] Chaurasia A., Gopal N., Firouzabadi F.D., Anari P.Y., Wakim P., Ball M.W., Jones E.C., Turkbey B., Huda F., Marston Linehan W. (2023). Role of Ultra-High b-Value DWI in the Imaging of Hereditary Leiomyomatosis and Renal Cell Carcinoma (HLRCC). Abdom. Radiol..

[137] Toro J.R., Nickerson M.L., Wei M.H., Warren M.B., Glenn G.M., Turner M.L., Stewart L., Duray P., Tourre O., Sharma N. (2003). Mutations in the Fumarate Hydratase Gene Cause Hereditary Leiomyomatosis and Renal Cell Cancer in Families in North America. Am. J. Hum. Genet..

[138] Zbar B., Tory K., Merino M., Schmidt L., Glenn G., Choyke P., Walther M.M., Lerman M., Linehan W.M. (1994). Hereditary Papillary Renal Cell Carcinoma. J. Urol..

[139] Zbar B., Glenn G., Lubensky I., Choyke P., Walther M.M., Magnusson G., Bergerheim U.S., Pettersson S., Amin M., Hurley K. (1995). Hereditary Papillary Renal Cell Carcinoma: Clinical Studies in 10 Families. J. Urol..

[140] Yang Y., Ricketts C.J., Vocke C.D., Killian J.K., Padilla-Nash H.M., Lang M., Wei D., Lee Y.H., Wangsa D., Sourbier C. (2021). Characterization of Genetically Defined Sporadic and Hereditary Type 1 Papillary Renal Cell Carcinoma Cell Lines. Genes. Chromosomes Cancer.

[141] Schmidt L., Duh F.M., Chen F., Kishida T., Glenn G., Choyke P., Scherer S.W., Zhuang Z., Lubensky I., Dean M. (1997). Germline and Somatic Mutations in the Tyrosine Kinase Domain of the MET Proto-Oncogene in Papillary Renal Carcinomas. Nat. Genet..

[142] PubMed Met, Metastasi, Motilità e Altro. https://pubmed.ncbi.nlm.nih.gov/14685170/.

[143] Birchmeier W., Brinkmann V., Niemann C., Meiners S., DiCesare S., Naundorf H., Sachs M. (1997). Role of HGF/SF and c-Met in Morphogenesis and Metastasis of Epithelial Cells. Ciba Found. Symp..

[144] Webster B.R., Gopal N., Ball M.W. (2022). Tumorigenesis Mechanisms Found in Hereditary Renal Cell Carcinoma: A Review. Genes.

[145] Novel Germline MET Pathogenic Variants in French Patients with Papillary Renal Cell Carcinomas Type I—Sebai—2022—Human Mutation—Wiley Online Library. https://onlinelibrary.wiley.com/doi/10.1002/humu.24313.

[146] Maher E.R. (2018). Hereditary Renal Cell Carcinoma Syndromes: Diagnosis, Surveillance and Management. World J. Urol..

[147] Macher-Goeppinger S., Keith M., Endris V., Penzel R., Tagscherer K.E., Pahernik S., Hohenfellner M., Gardner H., Grüllich C., Schirmacher P. (2017). MET Expression and Copy Number Status in Clear-Cell Renal Cell Carcinoma: Prognostic Value and Potential Predictive Marker. Oncotarget.

[148] Twardowski P.W., Tangen C.M., Wu X., Plets M.R., Plimack E.R., Agarwal N., Vogelzang N.J., Wang J., Tao S., Thompson I.M. (2017). Parallel (Randomized) Phase II Evaluation of Tivantinib (ARQ197) and Tivantinib in Combination with Erlotinib in Papillary Renal Cell Carcinoma: SWOG S1107. Kidney Cancer.

